# *Yersinia pseudotuberculosis* secretes an Fe (II)-binding effector to evade calprotectin-mediated nutritional immunity

**DOI:** 10.1007/s44154-026-00304-6

**Published:** 2026-04-14

**Authors:** Qingyun Dai, Hongxin Guan, Jianan Huang, Jing Hou, Mengsi Zhang, Yudi Wang, Pengfei Zhang, Lei Xu, Huawei Gu, Yao Wang, Songying Ouyang, Xihui Shen

**Affiliations:** 1 State Key Laboratory for Crop Stress Resistance and High-Efffciency Production, Shaanxi Key Laboratory of Agricultural and Environmental Microbiology, College of Life Sciences, Northwest A&F University, Yangling, Shaanxi 712100 P.R. China; 2 The Key Laboratory of Innate Immune Biology of Fujian Province, Provincial University Key Laboratory of Cellular Stress Response and Metabolic Regulation, Biomedical Research Center of South China, Key Laboratory of OptoElectronic Science and Technology for Medicine of the Ministry of Education, College of Life Sciences, Fujian Normal University, Fuzhou, 350117 China

**Keywords:** Type VI secretion system (T6SS), Ferrous iron transportation, Calprotectin, Nutritional immunity, OmpF, Oxidative stress, Acid stress

## Abstract

Iron is an essential cofactor for core metabolic processes and is critical to both host physiology and invading pathogens. While the competition between host and pathogen for ferric iron [Fe (III)] and heme has been well characterized, microbial strategies to overcome Fe (II) limitation—particularly under calprotectin (CP)-mediated Fe (II) chelation—remain poorly understood. In this study, we show that *Yersinia pseudotuberculosis* (*Yptb*) employs its type VI secretion system 1 (T6SS1) to acquire Fe (II) through secretion of the Fe (II)-binding effector SfeP. Deletion of *sfeP* significantly reduced bacterial loads in wild-type mice but not in CP-deficient mice, highlighting its essential role in virulence under CP-imposed Fe (II) restriction. Mechanistically, SfeP acts as a proteinaceous ferrousophore that specifically interacts with the outer-membrane porin OmpF to facilitate Fe (II) uptake, and the resulting SfeP-mediated iron homeostasis contributes critically to bacterial resistance against oxidative and acidic stress. Together, these findings uncover a T6SS-dependent Fe (II)-scavenging pathway in which SfeP cooperates with OmpF to counteract host nutritional immunity and promote *Yptb* virulence. This work not only underscores the versatility of T6SS in metal acquisition and stress adaptation, but also highlights the physiological significance of CP-mediated Fe (II) sequestration in host defense against bacterial infection.

**Supplementary Information:**

The online version contains supplementary material available at 10.1007/s44154-026-00304-6.

## Introduction

As a necessary cofactor for many enzymes and regulatory proteins, iron is the most commonly used transition metal in biology (Schaible and Kaufmann 2004). Its capacity to change between ferrous [Fe (II)] and ferric [Fe (III)] states, which permits electron transfer and redox catalysis, accounts for its versatility (Sousa Gerós et al. 2020). Ferric iron is more common in oxygen-rich environments, but its poor solubility restricts its availability. In anaerobic and low-pH environments, it can be converted into more soluble ferrous iron (Straub et al. 2001). Therefore, under aerobic conditions, biologically accessible iron is still rare even though it is abundant in the Earth's crust (Wandersman and Delepelaire 2004). As part of nutritional immunity, high-affinity binding proteins like ferritin, transferrin, lactoferrin, and hemoglobin further limit the availability of iron in hosts (Hood and Skaar 2012). Recent research has demonstrated that Fe (II) can also be effectively sequestered, particularly by calprotectin (CP), which binds Fe (II) with high affinity, although the majority of studies have concentrated on Fe (III) withholding (Nakashige et al. 2015).

With several binding sites for divalent metal cations, CP, which is made up of the S100A8 and S100A9 calcium-binding subunits, is an important component of the host's nutritional immunity system (Corbin et al. 2008). As one of the most abundant antimicrobial proteins in neutrophils, making up approximately 40% of their cytoplasmic protein content (Teigelkamp et al. 1991), CP is promptly released into the extracellular milieu upon infection (Liu et al. 2012). There, it functions by chelating essential trace metals, thereby restricting nutrient access to invading pathogens. While Mn (II) and Zn (II) sequestration is the most well-known function of CP, recent research has also demonstrated high-affinity binding to Fe (II), a mechanism that was previously overlooked. By limiting Fe (II) availability, CP can trigger iron starvation responses in diverse bacteria and thereby strengthen host defense (Nakashige et al. 2015). Nevertheless, it is still unclear what the wider physiological significance of CP-mediated Fe (II) withholding during infection is.

To acquire iron in the iron-restricted host environment, pathogenic bacteria deploy diverse strategies to evade nutritional immunity, including siderophore-mediated ferric iron uptake (Schalk 2025), hemophore-dependent heme capture (Krieg et al. 2009), and receptor-mediated acquisition of host iron-binding proteins such as transferrin (Je et al. 2025), lactoferrin (Noinaj et al. 2013), and hemoglobin (Ghigo et al. 1997). In low-oxygen and acidic niches where Fe (II) predominates, bacteria instead rely on ferrous iron uptake (Skaar 2010): Fe (II) is thought to diffuse across the outer membrane via porins and is subsequently imported through inner-membrane transporters such as FeoABC (Perry et al. 2007), YfeABCD (Katoh et al. 2001), MntH (Makui et al. 2000), and ZupT (Grass et al. 2005). Although Fe (II) competition has long been underappreciated, recent studies indicate that Fe (II) is abundant at infection sites and supports bacterial virulence (Lau et al. 2016). In particular, Fe (II) is essential for enteric pathogens in the anoxic intestinal tract, as *feoB* mutants of *Salmonella enterica* serovar Typhimurium (Kim et al. 2013), *Helicobacter pylori* (Velayudhan et al. 2000), and *Campylobacter jejuni* (Naikare et al. 2006) show impaired colonization. Although Fe (III) and heme acquisition are well understood, little is known about how pathogens get around CP-mediated Fe (II) sequestration.

The type VI secretion system (T6SS) is a conserved protein translocation apparatus widely distributed in Gram-negative bacteria that delivers effectors into both eukaryotic and prokaryotic cells (Ho et al. 2014; Gallegos-Monterrosa and Coulthurst 2021; Lin et al. 2021). Beyond its established roles in pathogenesis (Pukatzki et al. 2007; Jiang et al. 2014; Zhu et al. 2021; Song et al. 2025), bacterial competition (Russell et al. 2011; Basler et al. 2013; Zhao et al. 2018; Ma et al. 2014; Trunk et al. 2018; Zhu et al. 2026; Luo et al. 2023), biofilm formation (Yang et al. 2022) and stress resistance (Weber et al. 2009; Zhang et al. 2013), a subset of T6SSs contributes to metal acquisition by secreting specialized metal-binding effectors (Yang et al. 2021; Si et al. 2017b, a; Zhu et al. 2021; Han et al. 2019). However, a T6SS-secreted effector that directly binds ferrous iron (Fe^2+^) to actively promote Fe^2+^ uptake has not been reported. Here, we show that *Yersinia pseudotuberculosis* T6SS1 facilitates iron acquisition by secreting SfeP, a high-affinity Fe^2+^-binding effector that mitigates oxidative and acidic stress and counteracts calprotectin-mediated ferrous iron limitation in the intestinal environment. These findings reveal a previously unrecognized T6SS-dependent “ferrousophore”-mediated iron acquisition mechanism.

## Results

### The T6SS1 is required for stress defense in *Yptb*

**Fig. 1:**
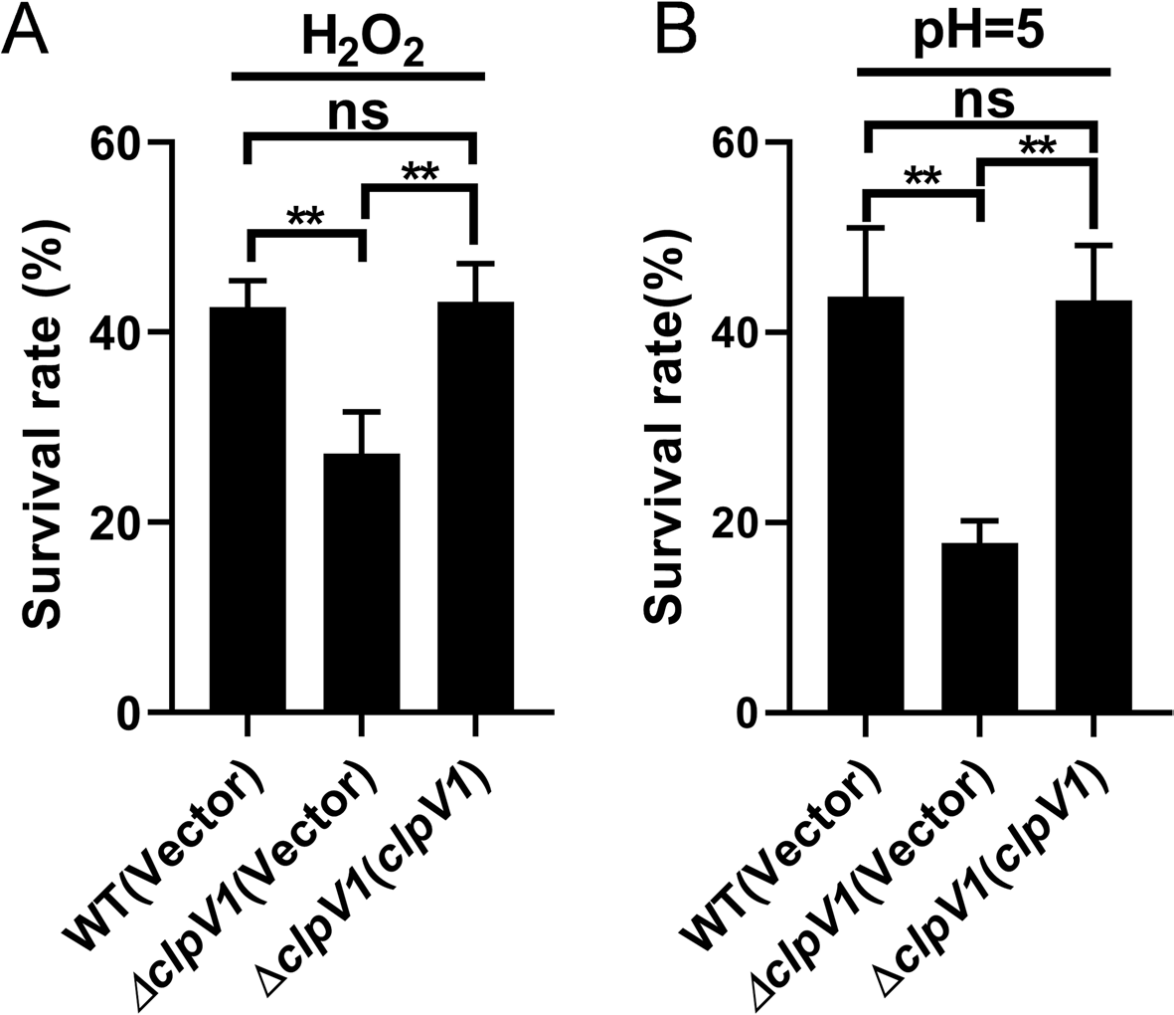
The T6SS1 is required for stress defense in *Yptb*. The *Yptb* WT strain, ∆*clpV1* mutant or complemented strain ∆*clpV1* (*clpV1*) grown to the stationary phase were exposed to H_2_O_2_ (1 mM) for 30 min (**A**) or exposed to acidic conditions (pH = 5) for 30 min (**B**) and the viability of the cells was determined. Error bars represent ± SEM. Statistical significance was assessed by one-way ANOVA with Tukey’s multiple-comparisons test. **P < *0.01*; ns, not significant

To determine whether T6SS1 contributes to environmental stress resistance in *Y. pseudotuberculosis*, we used ClpV1, the conserved AAA^+^ ATPase that powers sheath recycling and is essential for T6SS1 function, as a genetic marker to inactivate this system (Pietrosiuk et al. 2011). We compared the survival of the wild-type strain and the ∆*clpV1* mutant (T6SS1-deficient strain) under oxidative and acidic stress conditions. As shown in Fig. 1A, the *clpV1*-deficient strain exhibited significantly reduced survival when exposed to oxidative stress via hydrogen peroxide (H_2_O_2_) treatment. This sensitivity was reversed by genetic complementation. Similarly, the ∆*clpV1* mutant showed increased susceptibility to acidic stress (pH 5.0) in comparison to the WT strain; this phenotype was again mitigated by reintroducing the functional gene (Fig. 1B). Together, these results show that T6SS1 contributes significantly to *Yptb* resistance to oxidative and acidic stressors. Importantly, to exclude the possibility that the stress phenotypes arise from altered baseline fitness, we compared the growth of WT, ∆*clpV1*, and the complemented strain under non-stress conditions and observed no significant differences among the three strains (Fig. S1). Thus, the reduced survival of the ∆*clpV1* mutant under oxidative and acidic stress reflects a specific defect in stress resistance rather than impaired growth in standard conditions.

### T6SS1 secretes the ferrous iron-binding effector SfeP

**Fig. 2:**
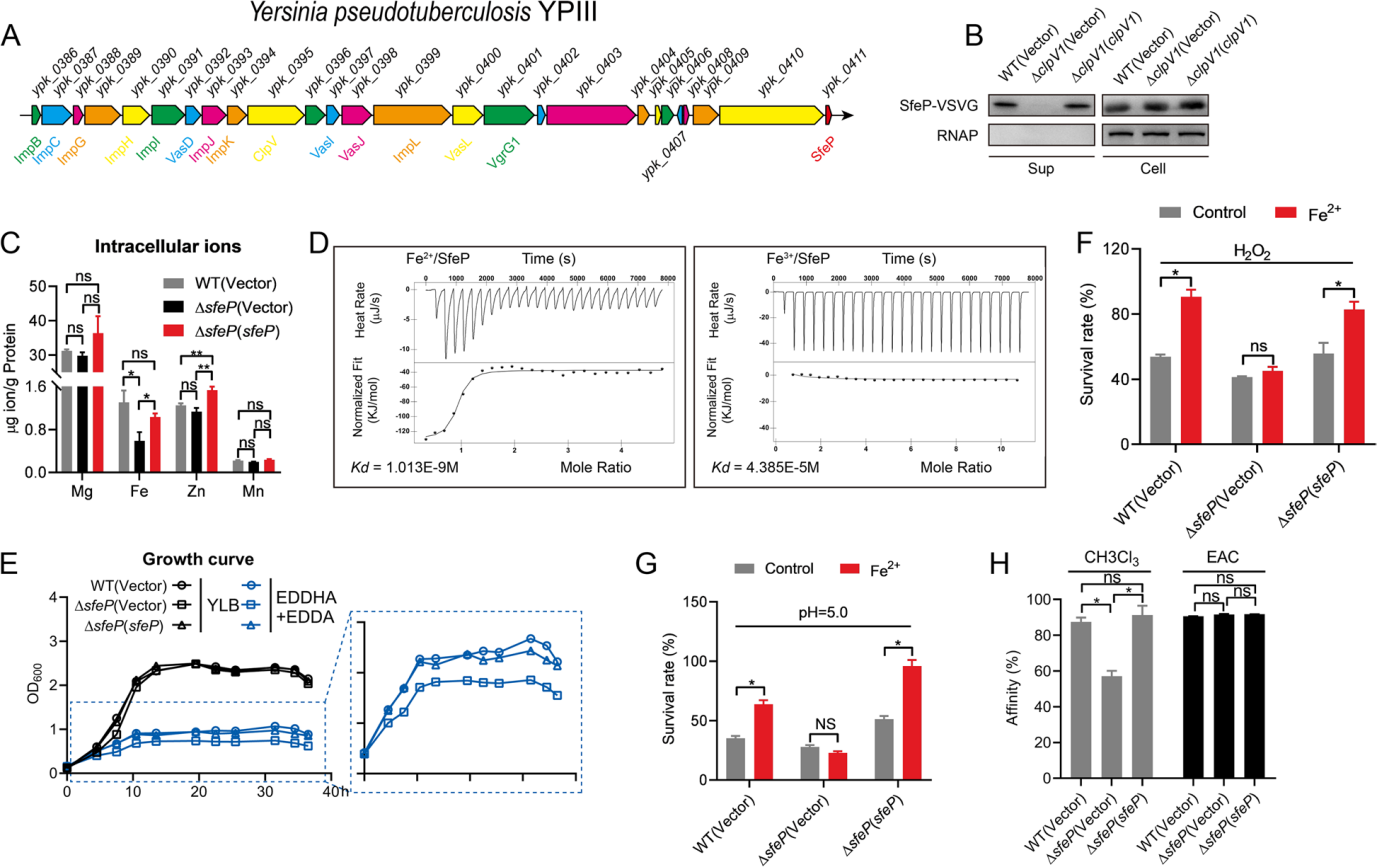
T6SS1 secretes a ferrous iron–binding effector SfeP. **A** Genetic organization of the *Yptb* T6SS1 locus; *sfeP* (*ypk_0411*) is indicated. **B** Immunoblot detection of SfeP in culture supernatants of the indicated strains. Cultures were grown in YLB to OD_600_ of 1.5. Whole-cell samples were prepared from 1 mL culture. Secreted proteins were collected from 120 mL 0.22-μm–filtered supernatants by three rounds of BA85 nitrocellulose membrane filtration and recovered in SDS sample buffer. Samples were normalized by culture OD_600_ and the processed volume (i.e., equal culture equivalents) prior to loading. Bacterial RNAP was used as the loading control. **C** Intracellular metal contents (Mg, Fe, Zn, and Mn) in WT, ∆*sfeP*, and complemented ∆*sfeP* (*sfeP*) strains were quantified by ICP-MS. **D** Binding of Fe (II) to SfeP measured by ITC. **E** Growth curves of WT, ∆*sfeP*, and complemented strains in YLB supplemented with 80 μM EDDHA and 6.5 mM EDDA, with or without 100 μM Fe (II). **F**, **G** Stationary-phase cultures were exposed to 1 mM H_2_O_2_ (30 min) (**F**) or acidic conditions (pH 5.0, 30 min) (**G**), with or without 100 μM Fe (II), and survival was quantified. **H** Cell surface electron donor/acceptor properties were assessed by measuring affinity to chloroform (CHCl_3_) and ethyl acetate (EAC) for mid-exponential-phase cultures. Error bars represent ± SEM. Statistical significance was assessed by one-way ANOVA with Tukey’s multiple-comparisons test. *P < *0.05*; **P < *0.01*; ns, not significant

Based on our previous studies demonstrating that the T6SS-4 of *Burkholderia thailandensis* secretes metal-binding effectors to acquire Mn (II) and Zn (II) for stress resistance (Si et al. 2017b, a), we hypothesized that the *Yptb* T6SS1 might similarly export proteins capable of binding metal ions. To identify candidate T6SS1-associated exported factors, we first performed a systematic annotation-guided scan of all predicted ORFs within the T6SS1 locus, with an emphasis on genes that (i) reside within the boundaries of the T6SS1 gene cluster (as defined by the conserved T6SS core components), (ii) are not annotated as canonical structural, regulatory, or housekeeping proteins, and (iii) encode small, hypothetical proteins, which are frequently enriched in secretion system loci as accessory proteins and/or specialized effectors. In addition, because cluster-terminal regions often harbor lineage-specific accessory genes, we prioritized uncharacterized ORFs located near the ends of the cluster to improve the likelihood of capturing system-specific secreted proteins. Screening the T6SS1 gene cluster identified YPK_0411, a 93-residue hypothetical protein located at the cluster terminus. Structural analysis revealed a degree of similarity between YPK_0411 and the iron-sulfur cluster-binding protein (PDB: 6FWR) (Fig. 2A and Fig. S2A-B). Phylogenetic analysis indicates that YPK_0411/SfeP homologs are broadly distributed across diverse bacterial taxa (Fig. S2C), suggesting that SfeP represents a conserved family of small proteins. Secretion assays using vesicular stomatitis virus G (VSVG)-tagged YPK_0411 detected the protein in WT culture supernatants but not in the ∆*clpV1* mutant (Fig. 2B), indicating T6SS1-dependent secretion. ICP-MS (inductively coupled plasma mass spectrometry) analysis showed significantly lower intracellular Fe levels in ∆*ypk_0411*, while Mg, Zn, and Mn remained unchanged, which is consistent with a role in iron acquisition (Fig. 2C).

Thus, we investigated if YPK_0411 directly binds iron. Using freshly prepared Fe (II) under oxidation-minimizing conditions, Fe (II) integrity was verified by ferrozine assays both before and after ITC (isothermal titration calorimetry) measurements. Background heats from metal-into-buffer control titrations were subtracted to correct for dilution and potential buffer–metal interactions. Under these conditions, ITC showed that YPK_0411 binds Fe (II) with high affinity (Kd = 1.013 × 10^–9^ M) but not Mg (II), Mn (II), Zn (II), Cu (II) or Ni (II) (Fig. 2D and Fig. S3A). Notably, YPK_0411 preferentially bound Fe (II) over Fe (III) (Fig. 2D), supporting its specific involvement in ferrous iron uptake. Based on these findings, we designated YPK_0411 as SfeP (T6SS-secreted ferrous iron-binding effector protein).

Functionally, the ∆*sfeP* mutant grew similarly to WT in nutrient-rich medium (Fig. S3B) but showed slower growth under Fe (II)-depleted conditions, including YLB supplemented with 80 μM EDDHA (ethylenediamine-N, N′-bis [2-hydroxyphenylacetic acid]) and 6.5 mM EDDA (ethylenediaminedi-[(o-hydroxyphenyl) acetic acid]) (Brickman and Armstrong 2012) (Fig. 2E). This defect was rescued by genetic complementation or excess Fe (II) supplementation (Fig. 2E), supporting a role for SfeP in ferrous iron acquisition. Furthermore, compared to the WT and complemented strains, the ∆*sfeP* mutant exhibited heightened susceptibility to H_2_O_2_ and acidic stress (Fig. 2F, G). This aligns with established evidence that iron homeostasis is critical for bacterial resistance to oxidative and acidic stress (Cornelis et al. 2011; Chen et al. 2020). Interestingly, exogenous Fe (II) significantly improved the survival of WT and complemented strains under these stress conditions but provided markedly less protection to the ∆*sfeP* mutant (Fig. 2F, G), consistent with impaired Fe (II) uptake in the absence of SfeP.

Because Fe (II) predominates in low-oxygen or acidic microenvironments, we further assessed cellular electron-donor/acceptor properties using chloroform (CHCl_3_) and ethyl acetate (EAC). The ∆*sfeP* mutant exhibited reduced binding to CHCl_3_ but not to EAC (Fig. 2H), consistent with a decreased intracellular pool of electron-donating species such as Fe (II). Together, these data indicate that the T6SS1 effector SfeP is required for efficient ferrous iron acquisition in *Yptb*, particularly under iron limitation and stress conditions.

### Structural features of SfeP and implications for metal binding

**Fig. 3:**
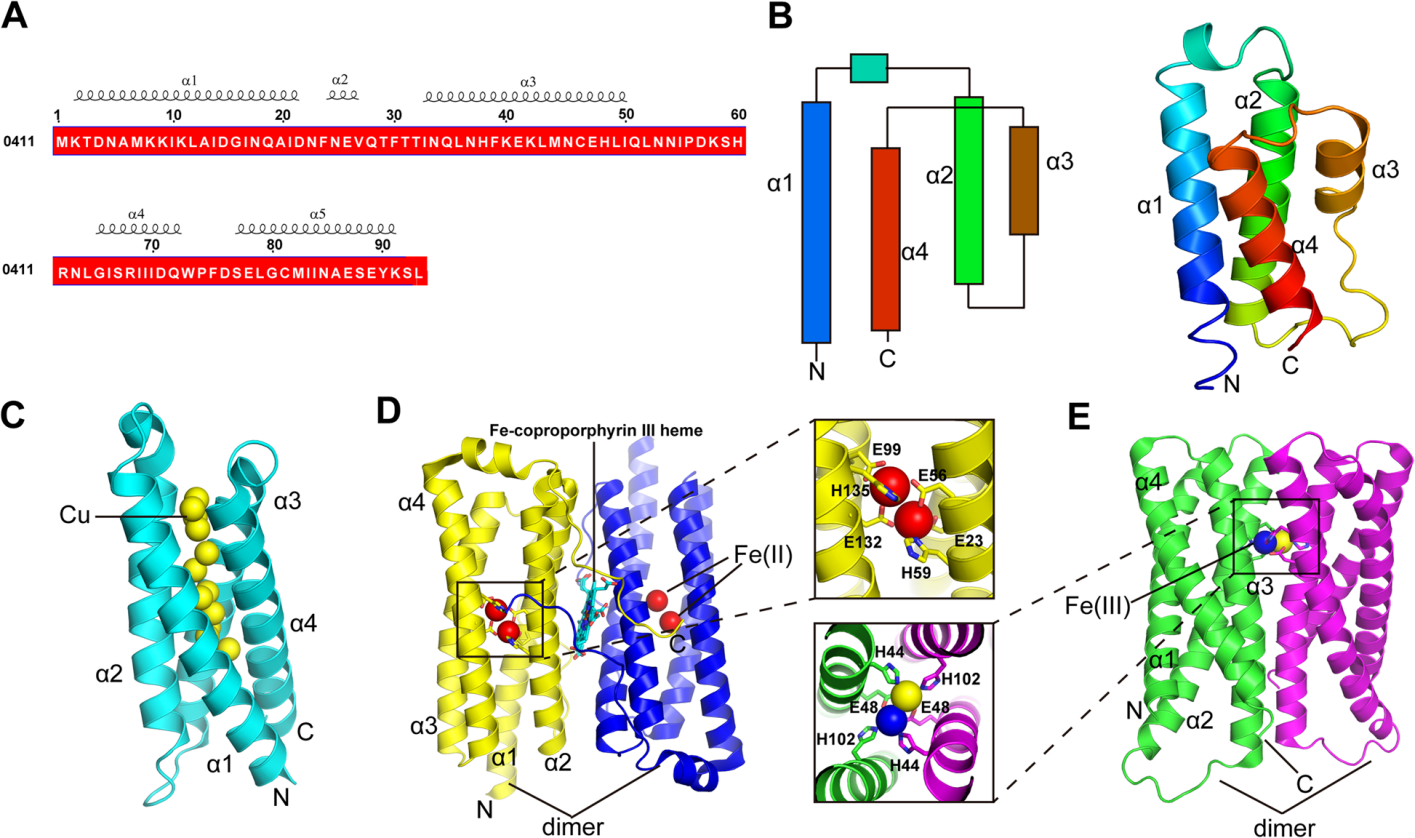
Overall structure of SfeP and structural comparisons with SlCsp3 and IMEF encapsulin cargo. **A** Amino acid sequence and secondary structure of SfeP. **B** Topology diagram (left) and overall structure of SfeP (right) (PDB: 7DMS). **C** Structure of SlCsp3 (PDB: 6Q6B) shown as a cartoon; bound copper ions are shown as yellow spheres. **D** Structure of ferritin with Fe-coproporphyrin III heme shown as cyan sticks; the di-iron centers in each monomer are shown as red spheres (PDB: 1NF4). **E** Di-iron ferroxidase site of the IMEF encapsulin cargo protein (PDB: 6N63); coordinating residues are shown as sticks, and the two ferric irons are shown as blue and yellow spheres

To provide structural insight into SfeP, we solved its crystal structure at 1.76 Å resolution in space group *P*2_1_2_1_2_1_ with one molecule per asymmetric unit (ASU) (Table. S1). The 93-residue effector adopts a four-helix bundle fold (Fig. 3A, B, PDB: 7DMS). Notably, no electron density corresponding to bound iron was observed in the apo-SfeP crystals, and we were unable to capture an Fe-bound complex under the crystallization conditions tested, potentially due to the oxidation sensitivity of Fe (II) and/or the transient nature of metal association. In addition, low and/or heterogeneous metal occupancy in the crystal lattice could further obscure metal features in electron density maps. Therefore, the structure presented here provides a framework for inferring potential metal-binding features, while biochemical assays (e.g., ITC) establish the Fe (II)-binding activity of SfeP. Future efforts to determine the Fe–SfeP complex structure will focus on anaerobic co-crystallization and rapid Fe (II) soaking using freshly prepared Fe (II) salts under reducing, oxygen-free conditions followed by immediate cryo-cooling, and, in parallel, cryo-EM will be explored as an alternative approach to capture potentially transient Fe-bound states. These efforts will be complemented by metal-substitution strategies (e.g., Ni (II)/Mn (II)) coupled with anomalous diffraction and XRF/ICP-MS verification of metal incorporation to enable unambiguous localization of the metal-binding site.

We compared the crystal structure of SfeP with all structures in the PDB using the Dali server (http://ekhidna.biocenter.helsinki.fi/dali_server/). The best hits which were related to metal ions binding protein were two Csps (copper storage proteins), *Sl*Csp3 (*Streptomyces lividans* copper storage proteins 3) and methanotroph *Methylosinus trichosporium* OB3b copper storage protein 1 (Csp1), which fold into similar four-helix bundle overall architecture (Vita et al. 2015) (Fig. 3C). The structural alignment of SfeP and *Sl*Csp3 gave the Z-score of 8.2 and gave the root-mean-square deviation (RMSD) of 2.7. SfeP and Csp1 have respective values of 7.4 and 2.9. However, unlike Csps that use the cysteine residues to hold the coppers within the four-helix bundle (Fig. 3C), no cysteine-rich motif was found in SfeP.

In addition, as the ubiquitous iron storage proteins, ferritins assemble into a spherical shell of 24 monomers, which then fold into four α-helix bundles. The di-iron center of each monomer employs particular residues such as glutamate and histidine to interact with ferrous iron (Fig. 3D). Although SfeP also adopts a four-helix bundle fold, it lacks the canonical ferroxidase motif and does not form the characteristic cage-like oligomer, suggesting a distinct mode of metal binding and handling. Similar to this, iron-mineralizing encapsulin-associated Firmicute (IMEF) proteins have a four-helix bundle structure, but they form dimers that use a different motif to coordinate two iron atoms at the subunit interface, creating a ferroxidase site (Fig. 3E) (Giessen et al. 2019). However, our structure did not show any dimeric SfeP. Notably, Fe (II) oxidation and/or dynamic association may have prevented us from capturing a Fe-bound SfeP complex. Future research using strictly anaerobic Fe (II)-supplemented conditions in conjunction with size-exclusion chromatography may help ascertain whether iron binding causes oligomerization or conformational changes in SfeP.

### SfeP affects biofilm formation and motility in *Yptb*

**Fig. 4:**
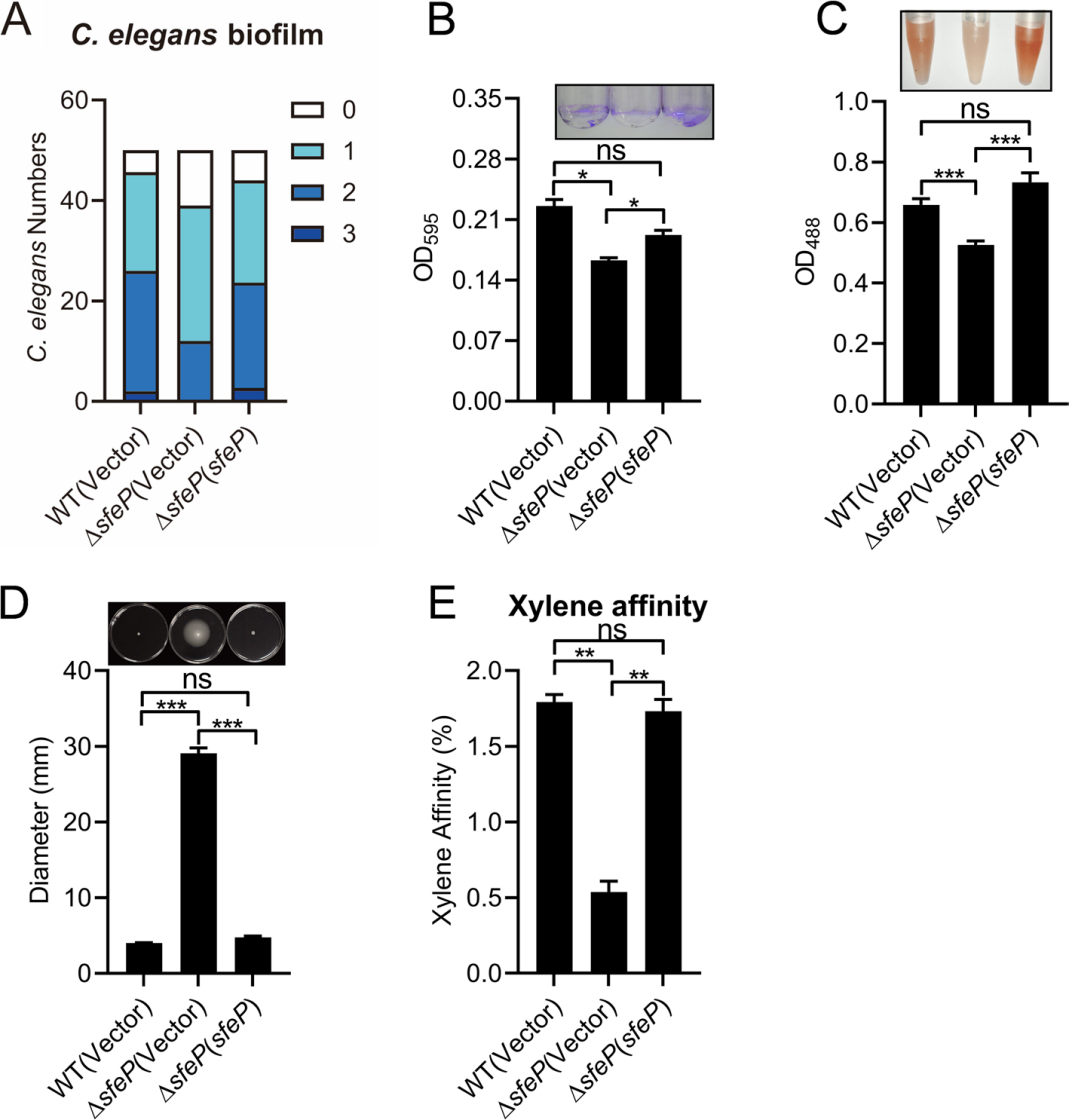
SfeP affects biofilm formation and motility in *Yptb*. **A** Biofilm severity on *C. elegans* infected with WT, ∆*sfeP*, or complemented ∆*sfeP* (*sfeP*) strains. **B** Biofilm formation on abiotic surfaces assessed by crystal violet staining. Representative staining is shown (top). **C** Extracellular polysaccharide production measured by Congo red staining. **D** Swimming motility assay on semi-solid agar plates. **E** Cell surface hydrophobicity measured by xylene affinity assay using mid-exponential-phase cultures. Error bars represent ± SEM. Statistical significance was assessed by one-way ANOVA with Tukey’s multiple-comparisons test. *P < *0.05*;**P < *0.01*; ***P < *0.001*; ns, not significant

Iron is essential for bacterial biofilm development, and ferrous iron [Fe (II)] has been implicated in promoting biofilm expansion (Oh et al. 2018). To assess whether SfeP contributes to biofilm formation, we used *Caenorhabditis elegans* as an in vivo surface model and scored nematode-associated biofilms after 24 h infection with GFP-expressing *Yptb* strains using a semi-quantitative 0–3 scale (S et al. 2011). In comparison to WT and the complemented strain, the ∆*sfeP* mutant displayed a higher percentage of low biofilm scores (levels 0–1) and a lower frequency of high scores (levels 2–3) (Fig. 4A), indicating impaired biofilm formation on living hosts. Consistently, biofilm biomass on abiotic surfaces was significantly reduced in ∆*sfeP* as measured by crystal violet staining (Fig. 4B), and Congo red staining revealed decreased extracellular polysaccharide production, a key biofilm matrix component (Darby 2008) (Fig. 4C). Because reduced biofilm formation is often accompanied by enhanced planktonic behavior, we further measured motility and found that deletion of *sfeP* markedly increased motility (Fig. 4D).

Additionally, bacterial surface hydrophobicity, which influences initial adhesion during biofilm establishment, was evaluated. The ∆*sfeP* mutant exhibited reduced hydrophobicity relative to the WT and complemented strains (Fig. 4E), further reinforcing the observed defects in biofilm formation. Collectively, these findings demonstrate that SfeP is indispensable for efficient biofilm formation in *Yptb* by modulating matrix composition and surface adhesion properties, and also impacts bacterial motility.

### SfeP is required for *Yptb* virulence

**Fig. 5:**
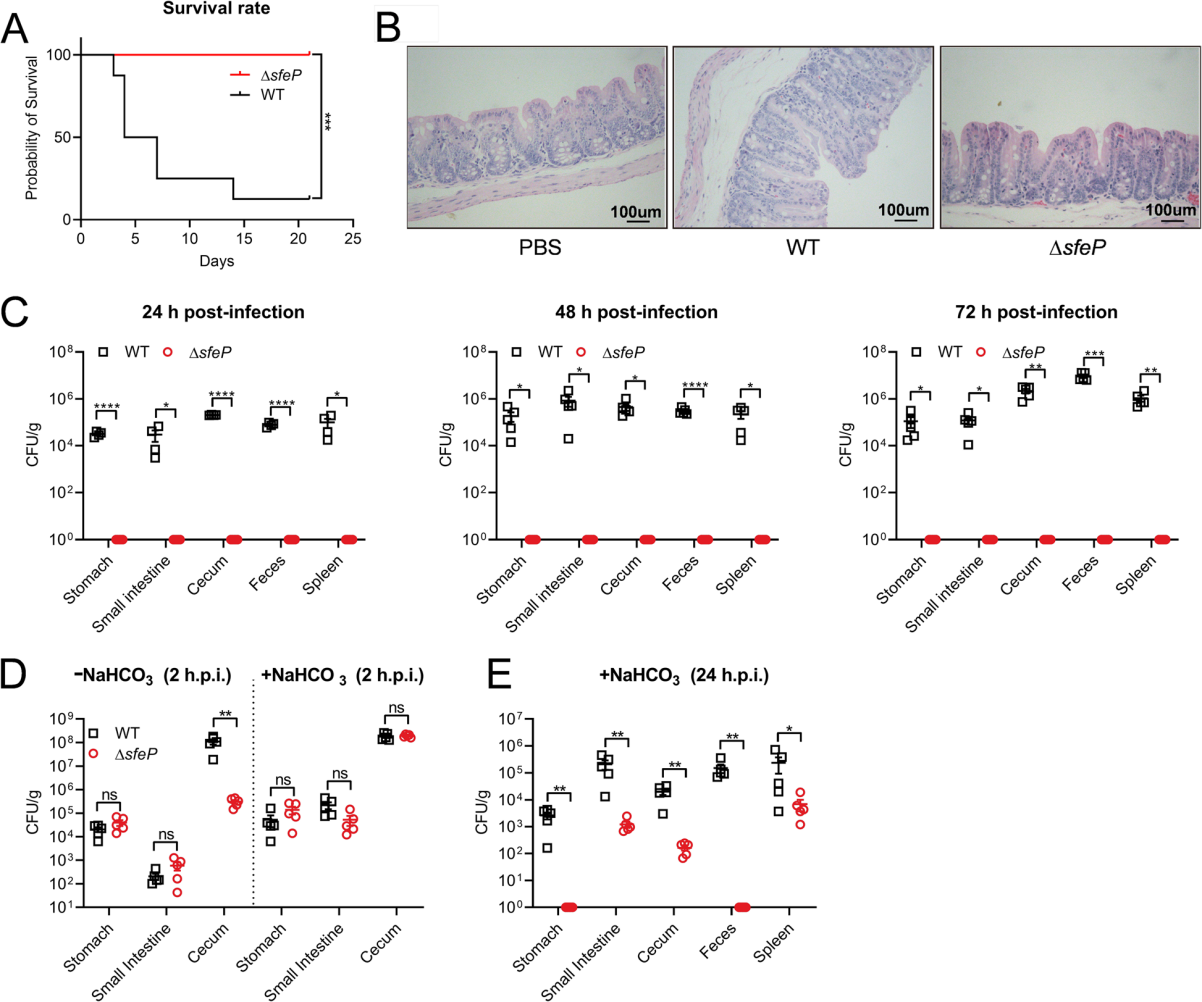
SfeP is required for *Yptb* virulence. **A** Survival of C57BL/6 mice after orogastric inoculation with WT or ∆*sfeP Yptb* (1 × 10^9^ CFU per mouse). **B** HE staining of cecum sections from infected mice at 72 h post-infection. **C** Bacterial burdens in the indicated organs at 24, 48, and 72 h after orogastric infection with WT or ∆*sfeP*. **D** Bacterial burdens at 2 h post-infection in mice left untreated or pretreated with 100 μL 5% NaHCO_3_ prior to orogastric infection. **E** Bacterial burdens at 24 h post-infection in mice pretreated with 100 μL 5% NaHCO_3_ prior to infection. Error bars represent ± SEM. Statistical significance was assessed by one-way ANOVA with Tukey’s multiple-comparisons test. *P < *0.05*;**P < *0.01*; ***P < *0.001*; ****P < *0.0001*; ns, not significant

Because iron acquisition is crucial for bacterial growth and virulence, we tested whether SfeP contributes to *Yptb* pathogenesis. C57BL/6 mice were orally infected with WT or ∆*sfeP* strains and monitored for survival. All ∆*sfeP*-infected mice survived to 21 days after infection, whereas less than 10% of WT-infected mice did (Fig. 5A), suggesting a significant attenuation of virulence. Histopathological analysis showed that WT-infected mice had severe intestinal damage, including mucosal abscission, epithelial disruption, and submucosal expansion; ∆*sfeP*-infected mice did not exhibit these pathological features (Fig. 5B); similar trends were seen in the spleen (Fig. S4). To assess whether this attenuation correlated with colonization defects, bacterial burdens were quantified in multiple tissues at 24, 48, and 72 h post-infection. WT bacteria colonized efficiently at all time points, whereas ∆*sfeP* bacteria were not recovered from the stomach, small intestine, cecum, spleen, or feces (Fig. 5C), supporting an essential role for SfeP in establishing infection.

We previously demonstrated that SfeP increases acid resistance (Fig. 2G), which is necessary for enteric pathogens to survive in the acidic stomach. To determine whether SfeP contributes to virulence beyond acid tolerance, mice were pretreated orally with NaHCO_3_ to neutralize gastric acidity and then infected with WT or ∆*sfeP Yptb*. Bacterial loads were similar in the two groups at 2 h after infection (Fig. 5D), and at 24 h, NaHCO_3_ significantly reduced ∆*sfeP* burdens in the spleen, cecum, and small intestine (Fig. 5E). In contrast, without NaHCO_3_ the ∆*sfeP* mutant showed a 3–5 log reduction in these tissues compared with WT and was undetectable in stomach samples and feces, suggesting impaired luminal persistence and/or fecal shedding with burdens below the detection limit. Overall, these data indicate that SfeP supports *Yptb* virulence by promoting acid stress survival and gut colonization, highlighting the importance of Fe (II) acquisition in the intestine.

### SfeP promotes bacterial fitness under CP-imposed Fe (II) restriction

**Fig. 6:**
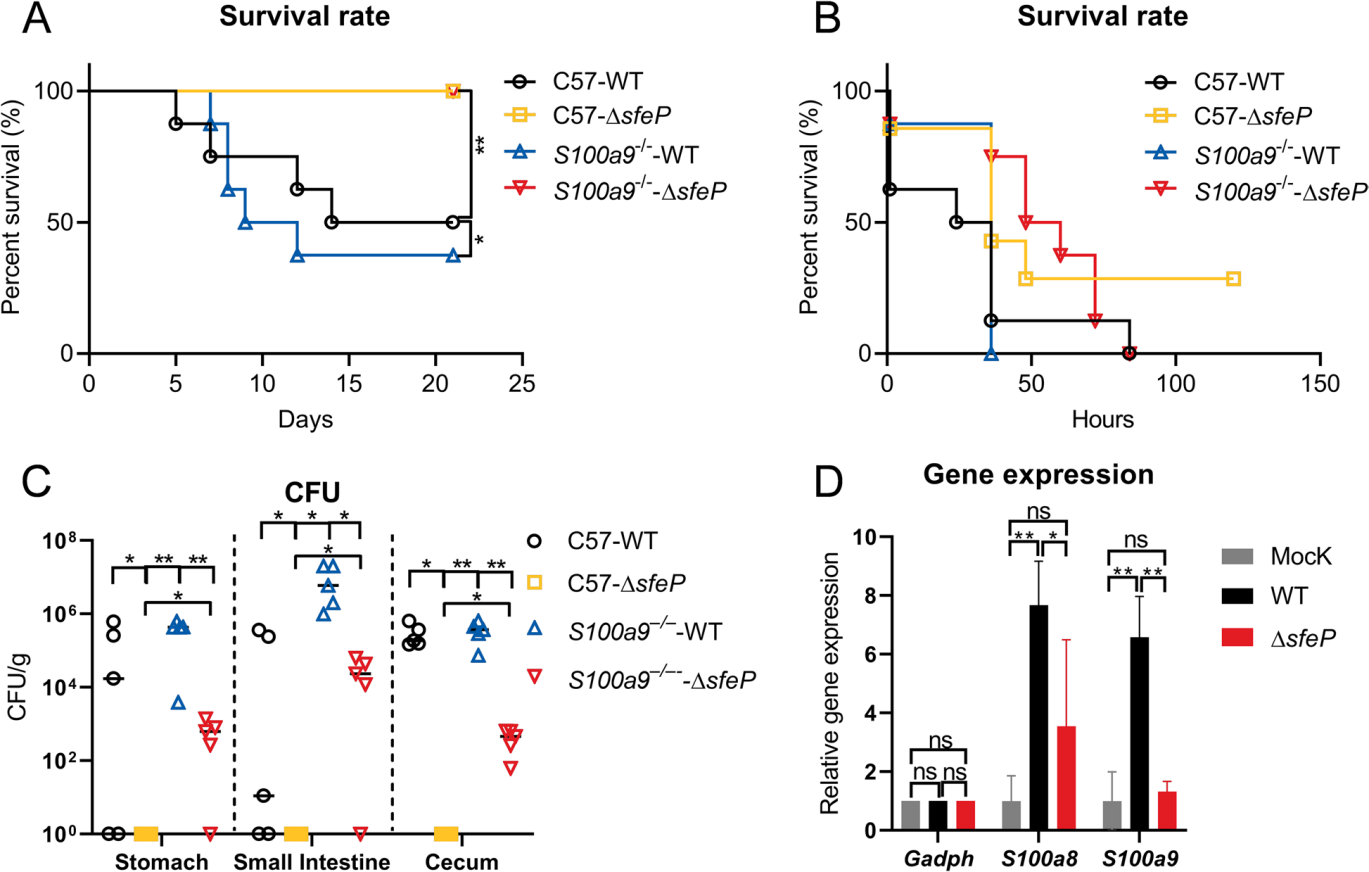
SfeP contributes to resistance against host calprotectin (CP)–mediated Fe (II) withholding. **A** Survival of wild-type C57BL/6 mice and calprotectin-deficient *S100a9*^*–/–*^ mice following orogastric infection with *Yptb* or Δ*sfeP* (3 × 10^9^ CFU per mouse). **B** Survival of C57BL/6 and *S100a9*^*–/–*^ mice following intraperitoneal infection with *Yptb* or Δ*sfeP* (1 × 10^7^ CFU per mouse). **C** Bacterial burdens (CFU) in the indicated organs at 40 h post-infection in C57BL/6 and *S100a9*^*–/–*^ mice infected with WT or ∆*sfeP* (3 × 10^9^ CFU). **D** qRT-PCR analysis of gene expression in C57BL/6-derived BMDMs infected with WT or ∆*sfeP* strains. Gene expression was normalized to a housekeeping gene and plotted relative to the indicated reference condition. Error bars represent ± SEM. Statistical significance was assessed by one-way ANOVA with Tukey’s multiple-comparisons test. *P < *0.05*; **P < *0.01*; ns, not significant

CP restricts pathogen growth by sequestering transition metals such as Mn and Zn (Zygiel and Nolan 2019), and recent studies have also implicated CP in limiting Fe (II) availability. To test whether SfeP-mediated Fe (II) uptake contributes to resistance against CP-dependent iron withholding, we infected C57BL/6 and CP-deficient (*S100a9*^*–/–*^) mice with WT or ∆*sfeP Yptb* (3 × 10^9^ CFU). *S100a9*^*–/–*^ mice were more vulnerable to WT *Yptb* than C57BL/6 mice following orogastric infection (Fig. 6A), indicating a protective role for CP; however, the effect of SfeP could not be determined because ∆*sfeP* was avirulent in both strains. Therefore, we used an intraperitoneal infection model, where ∆*sfeP*-infected *S100a9*^*–/–*^ mice showed significantly lower survival than ∆*sfeP*-infected C57BL/6 mice (Fig. 6B). At a dose of 1 × 10^7^ CFU (colony-forming units), approximately 25% of ∆*sfeP*-infected C57BL/6 mice survived to 120 h, whereas all *S100a9*^*–/–*^ mice succumbed by 80 h post-infection. These results indicate that CP-mediated nutritional immunity substantially shapes host susceptibility even when *sfeP* is absent, and that the infection outcome of the ∆*sfeP* mutant is strongly influenced by the host CP status.This conclusion was further corroborated by enumeration of the numbers of viable bacteria recovered from the small intestine, cecum and fecal at 40 h post intraperitoneal infection.

Consistently, although the ∆*sfeP* mutant showed severe colonization defects in C57BL/6 mice, these defects were largely reversed in *S100a9*^*–/–*^ mice, with high bacterial burdens recovered from intestinal tissues (Fig. 6C). This partial rescue suggests that CP contributes to the colonization disadvantage of the ∆*sfeP* mutant in vivo. In contrast, CP-deficient mice remained more susceptible to WT *Yptb* infection, as ∆*sfeP* still displayed a 2–3 log reduction in bacterial load relative to WT bacteria (Fig. 6C). Notably, the incomplete rescue of ∆*sfeP* in *S100a9*^*–/–*^ mice indicates that SfeP also promotes bacterial fitness through CP-independent mechanisms. Supporting these in vivo findings, expression of S100A8 and S100A9 was strongly induced in bone marrow–derived macrophages infected with WT *Yptb* but was substantially attenuated upon ∆*sfeP* infection (Fig. 6D), likely reflecting reduced bacterial fitness and host stimulation by the mutant. Together, these data support a model in which CP partially masks (or counterbalances) SfeP-dependent fitness advantages during infection: removal of CP partially alleviates the colonization defect of ∆*sfeP*, consistent with a role for SfeP in promoting Fe (II) acquisition under CP-associated Fe (II) limitation.

### SfeP transports ferrous iron by binding with the outer membrane porin OmpF

**Fig. 7:**
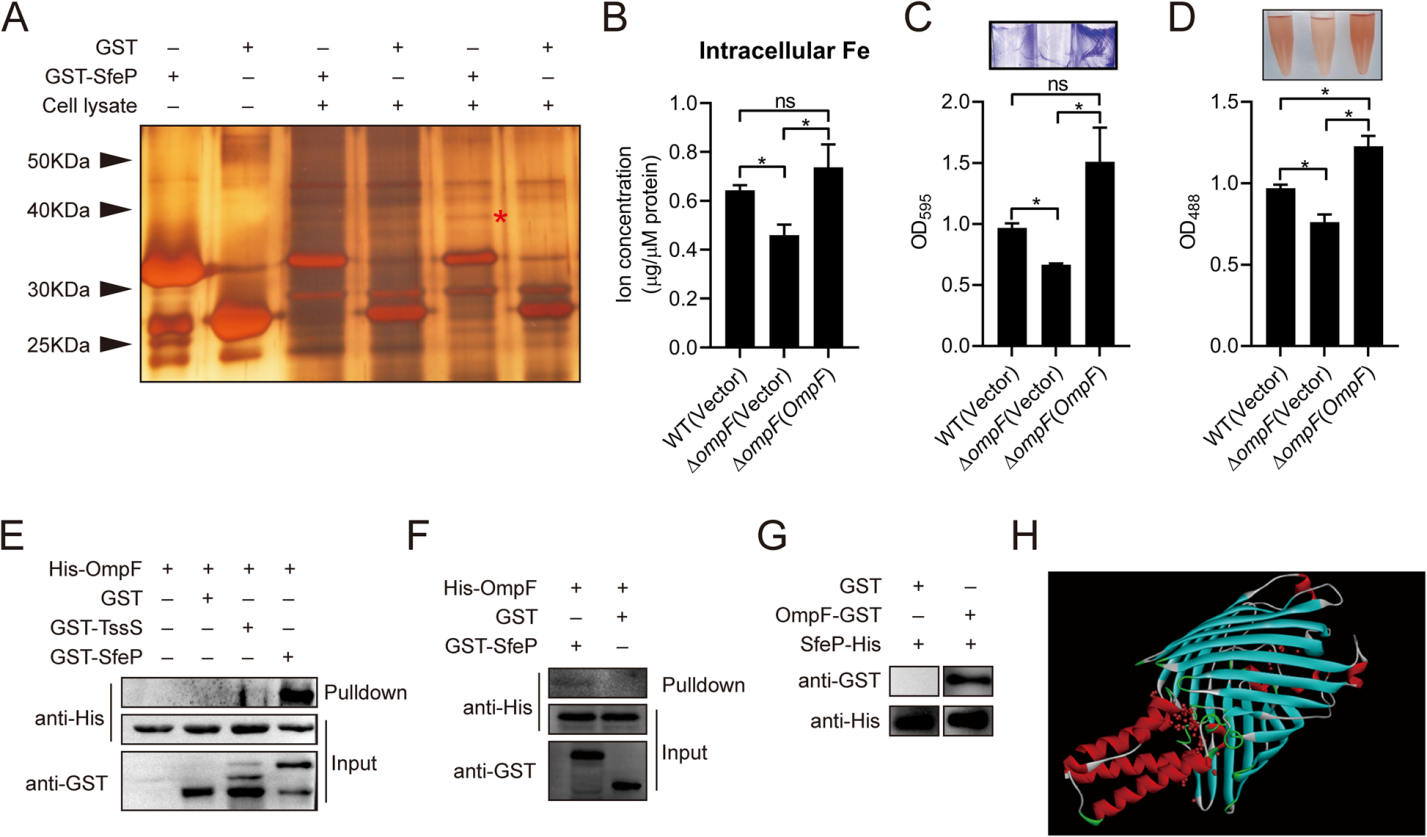
SfeP facilitates ferrous iron uptake by engaging the outer membrane porin OmpF. **A** GST pull-down identifying SfeP-interacting proteins from *Yptb* lysates or CHP-treated supernatants; the specific band (red asterisk) was analyzed by mass spectrometry. **B** Intracellular Fe levels in WT, ∆*ompF*, and complemented ∆*ompF* (*ompF*) strains measured by ferrozine assay. **C** Biofilm formation quantified by crystal violet staining after 20 h incubation in M9 medium. **D** Extracellular polysaccharide production measured by Congo red assay. **E**–**F** In vivo and in vitro GST pull-down assays confirming the SfeP–OmpF interaction by immunoblotting. His-OmpF (~ 38 kDa), GST-SfeP (~ 36 kDa), GST-TssS (~ 48 kDa). **G** Far-western assay showing binding of GST-OmpF to His-SfeP. GST-OmpF (~ 63 kDa). **H** Predicted docking model of SfeP with homology-modeled OmpF. Error bars represent ± SEM. Statistical significance was assessed by one-way ANOVA with Tukey’s multiple-comparisons test. *P < *0.05*; ns, not significant

It has been discovered that all known metal ion-binding T6SS effectors, such as TseM, YezP, and Azu, interact with outer membrane receptors to acquire metal ions (Si et al. 2017b; Wang et al. 2015; Han et al. 2019). To reveal how SfeP transports Fe (II) into the cell, we performed GST pull-down screening to identify such outer membrane receptors that mediate the Fe (II) transport activity of SfeP (Fig. 7A). The outer membrane porin OmpF (YPK_2649) was identified as a 40 kDa protein in the cell lysate that was specifically retained on GST-SfeP-coated beads. This protein exhibited a 57% amino acid sequence identity to the identified ferrous uptake porin OmpF in *E. coli* (Fig. S5A). Notably, a variety of bacteria contain the OmpF protein (Fig. S5B). To test the function of this *Yptb* OmpF in iron transport, we measured the iron concentrations in the WT, ∆*ompF* and complemented strains. Interestingly, both total iron and Fe (II) contents were significantly lower in the ∆*ompF* mutant while were completely restored to WT levels in the complemented strain, confirming the role of *Yptb* OmpF in Fe (II) acquisition (Fig. 7B). Moreover, similar to SfeP, the OmpF of *Yptb* was found to be involved in biofilm formation (Fig. 7C) and polysaccharide production (Fig. 7D).

The interaction between SfeP and OmpF was verified by performing in vivo GST pull-down assays. Western blotting reveals that GST-SfeP specifically binds to *Yptb* OmpF, but not the control proteins GST, GST-YezP, and GST-TssS (Fig. 7E). The specificity of this interaction was further confirmed through in vitro binding (Fig. 7F) and far-western assays (Fig. 7G) with purified GST-SfeP and His_6_-OmpF proteins. To explore a possible interaction mode, we predicted the structure of OmpF using I-TASSER and performed molecular docking analysis with ZDOCK. According to the resulting model, SfeP might bind close to OmpF's pore region (Fig. 7H). Together, these biochemical and modeling analyses indicate that SfeP directly interacts with OmpF and support a model in which SfeP engages the porin OmpF to promote SfeP-dependent Fe (II) uptake, potentially enabling the translocation of protein-complexed ferrous iron into the periplasm.

## Discussion

Iron plays a central role in bacterial growth and host–pathogen interactions (Nairz and Weiss 2020). Consequently, pathogens have evolved diverse strategies to acquire iron in its various chemical forms. Although the acquisition of ferric and heme-bound iron has been extensively studied (Hood and Skaar 2012), the mechanisms underlying ferrous iron [Fe (II)] uptake during infection remain comparatively understudied. In low-oxygen, acidic niches such as the hypoxic intestinal tract, Fe (II) often becomes a dominant and readily accessible iron source. It has been conventionally thought that Fe (II) passively diffuses across the outer membrane via porins (Perry et al. 2007). In contrast, our data reveal a previously unrecognized active Fe (II) acquisition system in which the secreted ferrousophore SfeP facilitates the transport of protein-complexed Fe (II) across the outer membrane through the porin OmpF. Phylogenetic analysis further indicates that SfeP homologs are distributed across diverse bacterial taxa (Fig. S2C), supporting an evolutionarily conserved role for this small-protein family. Unlike canonical Fe (II) transporters (e.g., Feo-type systems) that mediate direct membrane translocation, SfeP appears to function extracellularly by binding Fe (II) and promoting porin-dependent uptake, highlighting a mechanistically distinct route for Fe (II) acquisition. This active Fe (II) uptake mechanism meets cellular iron demand under host-mediated iron-withholding conditions during infection.

To combat bacterial infection, mammals deploy nutritional immunity, in which metal-sequestering proteins restrict the availability of essential transition metals (Hood and Skaar 2012). CP, an S100A8/S100A9 heterodimer best known for Mn (II) and Zn (II) scavenging (Zackular et al. 2015), also binds Fe (II) in vitro (Nakashige et al. 2015), though its physiological relevance in iron restriction has remained unclear. Here, we provide evidence that CP contributes to host defense against *Yptb* by competing for ferrous iron (Fig. 7), indicating that CP can inhibit microbial growth not only through Mn (II)/Zn (II) chelation but also via Fe (II) limitation. Beyond canonical transporters such as FeoABC (Lau et al. 2016), our data support a T6SS1-dependent, ferrousophore-mediated “Fe (II) mining” strategy in which the secreted effector SfeP enhances Fe (II) acquisition and counteracts CP-mediated restriction (Figs. 5, 6). Unlike FeoABC and other characterized Fe (II) transporters that primarily operate at the inner membrane to import free periplasmic Fe (II), the SfeP pathway provides an outer-membrane–proximal acquisition step. Specifically, SfeP captures extracellular Fe (II) and promotes its entry across the outer membrane via OmpF, thereby increasing Fe (II) availability for downstream transport under CP-imposed limitation (Perry et al. 2007). Together, these findings broaden our understanding of T6SS-dependent metal uptake and highlight CP-Fe (II) competition as a potential target for anti-*Yersinia* intervention.

The SfeP-mediated Fe (II) uptake pathway is conceptually analogous to the HasA hemophore system, in which the secreted heme-binding protein HasA captures heme and delivers it to the outer membrane receptor HasR for TonB-dependent uptake (Caillet-Saguy et al. 2009). Similarly, SfeP is secreted by T6SS1, binds extracellular Fe (II), and engages the porin OmpF to promote ferrous iron import. Multiple lines of evidence support the model that SfeP functions as a proteinaceous ferrousophore facilitating OmpF-mediated Fe (II) uptake: SfeP binds Fe (II) specifically but not Fe (III) (Fig. 2D); deletion of *sfeP* reduces intracellular iron accumulation (Fig. 2C); exogenous Fe (II) fails to protect the ∆*sfeP* mutant from oxidative and acidic stress (Fig. 2F, G); and SfeP directly interacts with OmpF (Fig. 7). Given that proteinaceous metallophores such as nickelophores (Cherrier et al. 2008) and zincophores (Łoboda and Rowińska-Żyrek 2017) have been described, we propose the term “ferrousophore” for this secreted Fe (II)-binding protein. Collectively, these findings reveal a previously unrecognized strategy for active ferrous iron acquisition.

T6SS has emerged as a versatile machinery for metal ion acquisition across bacterial species. Previous studies have established its role in scavenging Mn (II), Zn (II), and Cu (II) through secreted metal-binding effectors (Yang et al. 2021; Si et al. 2017b, a; Zhu et al. 2021; Han et al. 2019). In addition, T6SS contributes to ferric iron [Fe (III)] acquisition by secreting effectors that promote uptake of outer membrane vesicle (OMV)-associated iron (Lin et al. 2017; Li et al. 2022). More recently, a proteinaceous siderophore secreted by T6SS was reported to bind Fe (III) and mediate interbacterial competition (Song et al. 2024). Our discovery that *Yptb* T6SS1 mediates Fe (II) acquisition expands the known repertoire of T6SS metal transport functions and underscores several broader implications. The ability to acquire iron in multiple oxidation states enhances bacterial survival in dynamic host environments, where iron speciation varies across niches (e.g., microaerobic intestines vs. aerobic tissues). By deploying diverse iron acquisition strategies, pathogens can more effectively circumvent host nutritional immunity. The repeated emergence of T6SS-dependent metal uptake systems across taxa highlights the evolutionary pressure to maintain metal homeostasis in competitive and stressful environments, further positioning T6SS as a key player in bacterial resource competition and niche adaptation. Understanding the mechanistic diversity of T6SS-mediated iron acquisition could inform the development of anti-virulence strategies that target metal scavenging pathways without exerting broad-spectrum selective pressure.

## Conclusion

In summary, this study demonstrates that the T6SS1 of *Yptb* mediates ferrous iron acquisition through secretion of the effector SfeP, which specifically binds Fe (II) and enables the pathogen to evade calprotectin-mediated nutritional immunity. These findings add a new dimension to the growing paradigm of T6SS as a multifunctional metal-acquisition system and reinforce its critical role in bacterial stress adaptation, host colonization, and interbacterial competition. Furthermore, the widespread distribution of CP in host tissues suggests that targeting the CP–Fe (II) axis may offer a novel approach for developing therapeutic strategies against *Yersinia* infections.

## Materials and methods

### Ethical statement

All mice were on a C57BL/6 background. C57BL/6 mice were purchased from the Animal Center of Xi’An JiaoTong University (SCXK: Shan 2012–003, Xi’an, China). *S100a9*^*–/–*^ mice were purchased from Cyagen Inc.. Sex-matched male and female mice (6–12 weeks old) were housed under specific pathogen-free conditions.

### Bacterial strains and growth conditions

The bacterial strains used in this study are listed in Table. S2. *Yersinia pseudotuberculosis* YPIII (*Yptb*) and its derivatives were cultured at 26 °C with shaking (220 rpm) in *Yersinia*-Lysogeny Broth (YLB; 1% tryptone, 0.5% yeast extract, 0.5% NaCl, pH 7.0) or M9 minimal medium (6 g/L Na_2_HPO_4_, 3 g/L KH_2_PO_4_, 1 g/L NH_4_Cl, 0.5 g/L NaCl, 1 mM MgSO_4_, 0.1 mM CaCl_2_, 0.2% glucose) (Li et al. 2021).* E. coli* was grown in Luria–Bertani broth (LB; 1% tryptone, 1% yeast extract, 0.5% NaCl, pH 7.0) at 37 °C with shaking (220 rpm) and appropriate antibiotics. In-frame deletion mutants (∆*sfeP* and ∆*ompF*) were constructed by conjugation of WT *Yptb* with *E. coli* S17-1λ pir carrying pDM4-∆*sfeP* or pDM4-∆*ompF*, followed by selection on YLB agar containing nalidixic acid and chloramphenicol, counterselection on 20% sucrose, and confirmation by PCR (polymerase chain reaction) and DNA sequencing. To maintain plasmid, antibiotics were added using following concentrations: nalidixic acid (20 μg/mL), chloramphenicol (20 μg/mL), kanamycin (100 μg/mL), ampicillin (50 μg/mL).

### Growth curve assays

For growth curve measurements (e.g., Fig. 2E), overnight cultures were diluted to an initial OD_600_ (optical density at 600 nm) of 0.05 in the indicated media (YLB or M9) supplemented with the specified chelators and/or FeCl₂. Cultures were incubated at 26 °C with shaking (220 rpm), and growth was monitored by measuring optical density at OD_600_ at the indicated time points. Unless otherwise stated, all growth curve assays were performed with independent biological replicates, and data are presented as mean ± SEM.

### Ferrous iron supplementation and iron-limitation conditions

For ferrous iron supplementation assays, FeCl_2_ was used as the Fe (II) source. FeCl_2_ stock solutions were freshly prepared immediately before each experiment in deoxygenated water and added to cultures immediately prior to inoculation. FeCl_2_ was freshly prepared immediately before use and added immediately prior to inoculation to minimize oxidation. Where indicated, Fe (II)-limited conditions were established by supplementing the medium with EDDHA and/or EDDA (concentrations specified in the corresponding figure legends).

### Protein expression and purification

*E. coli* BL21 (DE3) cells harboring pET28a-sumo::*sfeP* were induced with 0.3 mM IPTG at OD_600_ of 0.8 and cultured at 18 °C for 16 h. Cells were harvested by centrifugation (4,500 rpm, 15 min, 4 °C) and resuspended in lysis buffer (50 mM Tris–HCl, pH 7.5, 150 mM NaCl). After sonication, lysates were clarified by centrifugation (17,000 rpm, 30 min, 4 °C) and the supernatant was applied to a Ni–NTA column (Qiagen). Resin-bound SUMO–SfeP was digested overnight with Ulp1 protease to remove the SUMO tag. Tag-free SfeP was eluted, concentrated, and further purified by size-exclusion chromatography on a Superdex 75 increase column (GE Healthcare) equilibrated with running buffer (25 mM HEPES, pH 7.5, 150 mM NaCl, 2 mM DTT).

### Crystallization, data collection and structural determination

Crystallization of SfeP (~ 25 mg/mL) was performed by hanging-drop vapor diffusion by mixing 0.6 μL protein with 0.6 μL reservoir solution. Diffraction-quality crystals were obtained at 16 °C within ~ 5 h in 0.1 M sodium malonate (pH 5.0) and 12% (w/v) PEG 3350. Crystals were harvested and flash-frozen in liquid nitrogen using 20% glycerol as a cryoprotectant. X-ray diffraction data were collected at beamline BL17U1 of the Shanghai Synchrotron Radiation Facility (SSRF), and processed with HKL-2000. The structure was solved by single-wavelength anomalous dispersion (SAD) using a selenomethionine-labeled crystal, and model building and refinement were carried out with COOT and PHENIX (Emsley et al. 2010; Adams et al. 2010). The final structure of SfeP was solved at 1.76 Å (Table. S1) and deposited into the Protein Data Bank (PDB) with the accession entry: 7DMS. The structure was analyzed by PyMol (http://www.pymol.org/).

### Plasmid construction

Plasmids and primers used in this study are listed in Table. S3 and S4. The in-frame deletion mutant ∆*sfeP* (*ypk_0411*) was generated using pDM4-∆*sfeP*: 918-bp upstream and 900-bp downstream flanking fragments were amplified with *sfeP*M1F_BglII/*sfeP*M1R and *sfeP*M2F*/sfeP*M2R_SalI, fused by overlap PCR, and cloned into pDM4. The ∆*ompF* (*ypk_2649*) deletion plasmid pDM4-∆*ompF* was constructed similarly using primers listed in Table. S4. For complementation, *sfeP* or *ompF* was amplified from *Yptb* genomic DNA with *sfeP*_FBamHI/*sfeP*_RSalI or *ompF*F_SalI/*ompF*R_BglII and inserted into pKT100 to generate pKT100-*sfeP* or pKT100-*ompF*; pKT100-*clpV1* was described previously (Wang et al. 2015). For recombinant expression, *sfeP* or *ompF* PCR products were digested with BamHI/SalI and cloned into pET28a or pGEX6p-1 to generate pET28a-*sfeP*, pET28a-*ompF*, pGEX6p-1-*sfeP*, and pGEX6p-1-*ompF*. To construct pME6032-*sfeP*-*vsvg*, *sfeP* was amplified with *sfeP*F-EcoRI/*sfeP*R-*vsvg*-TAA-XhoI, digested with EcoRI/XhoI, and ligated into pME6032. All constructs were verified by DNA sequencing.

### Protein secretion analysis

Secretion assays for SfeP (YPK_0411) was performed as described previously (Xu et al. 2014). Briefly, strains were grown in 150 mL YLB with appropriate antibiotics at 37 °C with shaking to OD_600_ of 1.5. For total protein controls, 1 mL of culture was pelleted and resuspended in 100 μL SDS loading buffer. For secreted proteins, 120 mL of culture was centrifuged, and the supernatant was filtered through a 0.22 μm membrane (Millipore, MA, USA). Secreted proteins in supernatant were collected by filtration over a nitrocellulose filter (BA85) (Whatman, Germany) for three times. The filter was then soaked in 100 μL SDS sample buffer for 20 min at 90 °C to recover the proteins. The OD_600_ of the culture and volume used in preparation were considered to normalize all samples.

### Isothermal Titration Calorimetry (ITC)

SfeP binding to metal ions was measured by ITC as described previously (Si et al. 2017b) using a Nano-ITC 2G (TA-Waters LLC, USA) at 25 °C in ITC buffer (20 mM Tris, pH 7.4, 150 mM NaCl, 10% glycerol, v/v). To minimize Fe (II) oxidation, Fe (II) solutions were freshly prepared immediately before each experiment from FeCl_2_ (or FeSO_4_) dissolved in deoxygenated Milli-Q water supplemented with 1 mM sodium ascorbate, and handled with minimal air exposure. Metal ions (1 mM; 250 μL syringe) were titrated into SfeP (50 μM; 1 mL cell) with 25 injections of 5 μL after baseline stabilization. Background heats (including dilution and potential buffer–metal interactions) were determined from metal-into-buffer control titrations and subtracted from the binding isotherms. Data were analyzed using Nano Analyze software (TA Instruments), and all experiments were performed in triplicate.

### Quantitative Real-Time PCR (qRT-PCR)

*Yptb* and derivatives were harvest at late-exponential phase shaking at 30 °C. RNA were extracted by using RNAprep Pure Cell/Bacteria Kit (TIANGEN, Beijing, China). RNA from mammalian cells was isolated with RNAeasy Animal RNA Isolation Kit with Spin Column (Beyotime Biotechnology, Haimen, China). RNA quality and concentration were assessed by agarose gel electrophoresis and NanoDrop spectrophotometry (Thermo Scientific). 500 ng of total RNA was converted into cDNA with FastKing RT Kit (With gDNase) (TIANGEN, Beijing, China). Quantitative real-time PCR (qRT-PCR) was performed on a CFX96 Real-Time PCR Detection System (Bio-Rad, USA) using TransStart Green qPCR SuperMix (TransGen Biotech, Beijing, China) under the following conditions: 95 °C for 10 min, followed by 40 cycles of 94 °C for 10 s and 50 °C or 58 °C for 30 s. Relative gene expression was normalized to *16S* rRNA (bacteria) or *gapdh* (mammalian cells) and calculated using the 2^−ΔΔCt^ method (Zuo et al. 2023). All samples were analyzed in triplicate, and qRT-PCR primers are listed in Table. S4.

### Bacterial survival assays

Mid-exponential phase *Yptb* strains grown in YLB medium were collected and diluted 50-fold into M9 medium and treated with H_2_O_2_ (1.0 mM) or supplied with or without 1 μM ferrous (FeCl_2_) for 35 min at 26 °C. After treatment, the cultures were serially diluted and plated onto YLB agar plates, and colonies were counted after 36 h of growth at 26 °C. Percentage survival was calculated by dividing the number of CFU of stressed cells by the number of CFU of cells without stress (Wang et al. 2015).

### Determination of intracellular ion content

Intracellular ion content was determined as described (Wang et al. 2015). Briefly, cells were grown in YLB to the post-exponential phase, and 20 mL cultures were harvested and washed twice with PBS. Pellets were lysed in BugBuster (Novagen, Madison, WI) for 12 h with rotation, and total protein was quantified using a NanoDrop ND-1000 spectrophotometer (Thermo Fisher Scientific). For ICP-MS, lysates were diluted 100-fold in 2% (v/v) trace-metal–grade nitric acid to a final volume of 5 mL and analyzed on an ICP-MS instrument (Varian 802-MS). Mg^2+^, Fe^2+^, Zn^2+^ and Mn^2+^ concentrations were quantified using external calibration curves prepared from certified multi-element standards, with acid-matched blanks subtracted; standards and samples were prepared in the same nitric acid matrix to minimize matrix effects. Metal contents were normalized to total protein and reported as ng metal per mg protein. Unless otherwise stated, measurements were performed with three independent biological replicates.

### Congo red assay

Relevant strains were cultured at 30 °C in M9 medium overnight. The bacterial cells were collected and washed using ddH_2_O three times. Cells were then resuspended in Congo red solution (0.4%) and incubated at 37 °C for 30 min. Nonspecifically bound Congo red was removed by washing with 1 M NaCl for 20 min, and the bacteria were washed with ddH2O three times. Cells were washed by ddH_2_O for three times and suspended in 1 mL ddH_2_O. The absorption wavelength for each sample was detected at 488 nm.

### GST pull-down assay

The GST pull-down assay was performed as described (Si et al. 2017b). Briefly, 0.5 mg purified GST fusion protein was incubated with cleared lysates from a 200 mL *Yptb* culture for 3 h at 4 °C, followed by addition of 100 μL prewashed glutathione beads and further incubation for 2 h. Beads were washed five times with PBS, and bound proteins were eluted with SDS sample buffer, resolved by SDS/PAGE, and visualized by silver staining (Bio-Rad). Selected bands were excised, trypsin-digested, and analyzed by MALDI-MS (matrix-assisted laser desorption/ionization/mass spectrometry) (Voyager-DESTR, Applied Biosystems). For in vivo pull-down, cells co-transformed with pET28a-*ompF* and pGEX6p-1-*sfeP* were harvested from ~ 1 L cultures and lysed. The clarified lysates were incubated with 200 μL prewashed glutathione beads at 4 °C for 3 h, followed by an additional 2 h incubation. Beads were washed five times with PBS, and retained proteins were detected by immunoblotting with an anti-His antibody (Millipore). Input lysates were probed with anti-His (Millipore) and anti-GST (Santa Cruz, USA) antibodies. For in vitro pull-down, purified GST-SfeP (or GST control) was incubated with His_6_-OmpF in PBS for 2 h at 4 °C, followed by addition of 40 μL prewashed glutathione bead slurry and incubation for an additional 2 h; beads were washed five times with TEN buffer (100 mM Tris–HCl, pH 8.0, 10 mM EDTA, 500 mM NaCl) and bound proteins were detected by anti-His immunoblotting. All immunoblotting-based experiments were performed with at least three independent biological replicates.

### Mouse infections

All animal experiments were conducted under the animal welfare assurance policy of Northwest A&F University. Mid-exponential phase *Yptb* strains grown in YLB at 26 °C were washed twice with sterile PBS and used for orogastric infection of 6–12-week-old female C57BL/6 mice using a ball-tipped feeding needle. For survival assays, mice were gavaged with 1 × 10^9^ of each strain and monitored daily for 21 days (Zhu et al. 2021). For intraperitoneal survival assays, mice were injected with 1 × 10^7^ CFU and monitored for 150 h. For bacterial burden analysis, fecal pellets were collected from individual mice at indicated time points, weighed, and homogenized in PBS. For tissue burdens, mice were euthanized by CO_2_ asphyxiation followed by cervical dislocation, and the cecum, colon, spleen, and liver were weighed, homogenized in PBS, serially diluted, and plated on YLB agar containing nalidixic acid (20 μg/mL). CFU were enumerated and expressed as CFU per gram of tissue.

### Hematoxylin and eosin (HE) staining

Different tissues were dissected, fixed in 10% (vol/vol) neutral buffered formalin (Sigma-Aldrich), and subsequently embedded in paraffin. Sections of 5-μm thickness were prepared and stained with hematoxylin and eosin (H&E). For each animal, single random sections were examined.

### Macrophage infection

To generate bone marrow derived macrophages (BMDMs), the primary marrow stromal cells were isolated from the femur of mice and cultured in RPMI 1640 medium supplemented with FBS (10%), penicillin (100 U/mL), streptomycin (100 μg/mL), 2-mercaptoethanol (50 μM), L-Glutamine (2 mM) and M-CSF (100 ng/mL) for 7 days (Tang et al. 2024). *Yptb* strains were grown overnight in YLB at 26 °C with appropriate antibiotics, harvested the next day, and resuspended in sterile PBS. BMDMs were cultured in RPMI 1640 medium without FBS and penicillin–streptomycin prior to infection. Cells were infected with *Yptb* at an MOI of 20 or 50 (Chung et al. 2016), centrifuged at 500 × g for 5 min to promote bacterial contact, and incubated at 37 °C for 2 h. After two PBS washes, fresh RPMI containing FBS and penicillin/streptomycin was added, and cells were further incubated at 37 °C with 5% CO₂ for 4 h. Cells were harvested at 6 h post-infection for RNA extraction (Tang et al. 2024).

### Far western blot assay

BL21 cells carrying pET28a-*sfeP* were cultured in 300 mL LB and induced with IPTG for 8 h. Cells were harvested, resuspended in 25 mL Tris–HCl buffer containing 1% Triton X-100, and lysed by sonication. Lysates were resolved by 12% SDS-PAGE and transferred onto PVDF membranes (Millipore). Membranes were blocked with 5% BSA for 6 h and then incubated with bait proteins (GST or OmpF-GST) at 4 °C for 8 h. Membranes were incubated with anti-His or anti-GST primary antibodies for 4 h, washed three times with TBST (50 mM Tris–HCl, 150 mM NaCl, 0.05% Tween 20, pH 7.4), and then incubated with HRP-conjugated secondary antibodies (Beyotime Biotechnology, China) for 1 h. Signals were visualized using an ECL kit (Invitrogen) according to the manufacturer’s instructions. All immunoblotting-based experiments were performed with at least three independent biological replicates.

### Ferrous and total iron quantification

Ferrous iron detection assay (ferrozine assay) was performed as described (Viollier et al. 2000). Briefly, 100 mL culture were collected and washed with PBS for twice. The cell pellet was lysed by using 1 mL Bugbuster (Novagen, Madison, WI) for 12 h with rotating incubation. Supernatant were collected to determine the iron concentrations. For detecting ferrous concentration, 200 μL supernatant was added with 20 μL ferrozine. For detecting total iron, 160 μL supernatant was added with 20 μL ferrozine solution, 30 μL hydroxylamine hydrochloride and 10 μL HAC-NH_4_AC. The absorption at 562 nm was obtained for each sample and the content of ferrous and total iron was calculated by using standard curve.

### Motility assay and biofilm formation assay

Swimming motility was assayed on semi-solid agar plates as described (Atkinson et al. 1999). Biofilm formation was measured using the test tube, performed as previously described (Guan et al. 2015). Briefly, *Yptb* strains were grown in YLB and inoculated into 3 mL M9 medium containing 0.4% glucose, then incubated at 26 °C with shaking (220 rpm). After 24 h, tubes were gently washed three times with PBS, stained with 1% crystal violet for 15 min, and washed three additional times with PBS. Bound crystal violet was solubilized with 95% ethanol, and absorbance at 595 nm was measured using a microplate reader (BioTek Instruments, Inc.). Biofilm formation on *C. elegans* was assayed as described (Tan and Darby 2004). Briefly, GFP-labeled *Yptb* strains were cultured overnight in YLB (30 °C, 220 rpm), and 1 mL of culture was spread onto NGM agar plates and incubated at 30 °C for an additional day. Biofilms were accumulated on about 50 nematodes per plate by placing *C. elegans* on *Yersinia* lawns and incubating them at 25 °C for 1 to 2 days. To identify the GFP probe, the worms were washed off with 10 mL buffer (0.01 M KH_2_PO4, 0.15 M NaCl, pH 7.3), pelleted (100 × g, 1 min), and washed twice more with 1 mL buffer to remove loosely associated bacteria. Fluorescence images were captured using a Leica DM5000B microscope (Leica Microsystems, Wetzlar, Germany), and biofilm coverage was scored on a 0–3 scale as described. Unless otherwise stated, assays were performed with three independent biological replicates, and data are presented as mean ± SEM.

### Bacterial cell surface hydrophobicity assay

Microbial adhesions to solvents were measured by the method described before (Wang et al. 2013). *Yptb* strains were grown in YLB broth and then transferred into 3 mL of M9 medium containing 0.4% glucose, shaking at 26 °C (220 rpm). Next, 2 mL culture medium was mixed with 2 mL xylene. The mixture was vortexed for 2 min and stood for 15 min. The absorption wavelength of the 600 nm water phase was obtained.

### Statistical analysis

Experimental data analyzed for significance were performed by using GraphPad Prism 6 (GraphPad Software, San Diego, California, USA). *P* values for mouse survival were calculated using the Log-rank (Mantel-Cox) test. *P* values for bacterial CFU in mouse tissues were calculated using the Mann–Whitney test (I). Error bars represent ± SEM. Statistical significance was assessed by one-way ANOVA with Tukey’s multiple-comparisons test. *P < *0.05*; **P < *0.01*; ***P < *0.001*; ****P < *0.0001*; NS, not significant.

## Supplementary Information

Supplementary Material 1. Supplementary Figures: Fig. S1 Growth curves of *Yptb* WT, ∆*clpV1* mutant or complemented strain ∆*clpV1*(*clpV1*). Saturated bacterial cultures were diluted to fresh YLB medium. The growth of the cultures was monitored at indicated time points by measuring OD_600_. Fig. S2 SfeP is a Fe binding protein. (A) Top 10 threading templates of YPK_0411 predicted by I-TASSER. I-TASSER modeling starts from the structure templates identified by LOMETS from the PDB library. (B) Part of the residues of YPK_0411 and 6fwrA, the residues are colored in black, those residues in template which are identical to the residue in the query sequence are highlighted in color. (C) Phylogenetic relationship of *Yptb* YPK_0411 with homologous proteins in other bacteria. Different protein sequences were obtained from the SwissProt database. The phylogenetic tree was constructed using MEGA 6.0 by the neighbor-joining method and multiple sequence alignment was performed using CLUSTAL W. The scale bar indicates percentage of divergence (distance). SwissProt accession nos. of proteins from species are as follows: *Xenorhabdus khoisanae* (WP 348994227.1:1-92); *Xenorhabdus khoisanae *(WP 053067915.1:1-87); *Xenorhabdus bovienii *(WP 275366913.1:1-94); *Y. pseudotuberculosis* (YPK_0411); *Y. pseudotuberculosis* (WP 032466985.1:1-87); *Salmonella enterica* (EOF5965430.1:18-90); *Pantoea ananatis* (WP 264239268.1:3-95); *Pantoea agglomerans* (WP 277971703.1:3-95); *Pseudomonas sp.* (WP 369319847.1:45-138); *Pseudomonas graminis* (WP 083233100.1:50-142); *Pseudomonas sp. *(WP 401361070.1:1-95); *Pseudomonas syringae* (WP 122259395.1:1-88); *Pseudomonas syringae pv. *(EEB57359.1:20-107); *Pseudomonas* (WP 005771784.1:9-95); *Methylococcales bacterium* (MGH8550762.1:35-92); *Agrobacterium vitis *(WP 070149548.1:30-88); *Myxococcota bacterium* (MFC1482338.1:1-89); *Streptomyces sp. *(WP 269859640.1:3-89); *Streptomyces sp.* (WP 355860097.1:8-45); *Streptacidiphilus jiangxiensis* (WP 042442920.1:3-96); *Streptomyces sp.* (WP 390857820.1:5-97); *Neisseria polysaccharea* (WP 304678009.1:1-93); *Streptococcus timonensis *(WP 414338493.1:1-93). Fig. S3 SfeP is a ferrous iron binding protein. (A) The binding of Mg (II), Mn (II) Zn (II), Cu (II) and Ni (II) with SfeP protein was determined via ITC. Representative raw thermograms (upper panels) and the corresponding integrated heat plots with best-fit curves (lower panels) are shown. Heats of dilution (metal titrated into buffer alone) were subtracted prior to fitting. (B) Growth curves of *Yptb* WT, ∆*sfeP* mutant or complemented strain ∆*sfeP* (*sfeP*). Saturated bacterial cultures were diluted to fresh YLB medium. The growth of the cultures was monitored at indicated time points by measuring OD_600_. Fig. S4 SfeP is required for *Yptb* virulence. Hematoxylin-Eosin (HE) staining of the spleen of the C57BL/6 mice orogastrically inoculated with *Yptb* WT strain or ∆*sfeP* mutant. Tissues was collected at 72 h post-infection. Fig. S5 The OmpF protein is widespread in bacteria. (A) Amino acid sequence similarity between *Escherichia coli* K-12 MG1655 OmpF (b0929) and *Yersinia pseudotuberculosis *OmpF (YPK_2649). The different residues are highlighted in red. (B) Phylogenetic relationship of *Yptb* OmpF with homologous proteins in other bacteria. Different protein sequences were obtained from the SwissProt database. The phylogenetic tree was constructed using MEGA 6.0 by the neighbor-joining method and multiple sequence alignment was performed using CLUSTAL W. The scale bar indicates percentage of divergence (distance). SwissProt accession nos. of proteins from species are as follows: *Y. pseudotuberculosis serotype* YPIII YPK_2649 (tr|A0A0H3B637); *Y. pestis* (tr|Q0WH04); *E. tribolii* (tr|A0A370QMI8); *X. mauleonii *(tr|A0A1I3I0B7); *X. koppenhoeferi* (tr|A0A1I7EVN7); *P. stewartii subsp. stewartii DC283 *(tr|H3RBC8); *X. cabanillasii *(tr|A0A3D9UH17); *Biostraticola tofi *(tr|A0A4R3Z2Q4); *S. praecaptivus* (tr|W0HUV4); *E. gerundensis* (tr|A0A0U5L440); *X. mauleonii* (tr|A0A1I3QI79); *K. pneumoniae subsp. Rhinoscleromatis* (tr|A0A378DHK5); *K. pneumoniae IS39* (tr|W1HX06); *B. agrestis ATCC 33320* (tr|A0A085GGU6); *R. electrica *(tr|A0A514ETS7); *X. koppenhoeferi* (tr|A0A1I7IDA7); *E. coli O6:K15:H31* (sp|P0DSD9); *E. cloacae S611* (tr|V5A844); *S. flexneri serotype 5a* (sp|A0A4P7TN82); *S. typhi* (sp|Q56113); *K. pneumoniae *(tr|W1DQ52); *C. Tachikawaea gelatinosa *(tr|A0A090BWJ9); *S*. typhimurium (strain LT2) (sp|P37432);* S.* typhimurium (strain SL1344) (sp|A0A0H3N9T8); *E. coli *(strain K12) (sp|P02931); *X. nematophila *(sp|Q56828); *S. marcescens* (sp|O33980). Fig. S6 The original images used in the manuscript. (2B) Immunoblot detection of SfeP in culture supernatants of the indicated strains. (4B) Biofilm formation on abiotic surfaces assessed by crystal violet staining. (4C) Extracellular polysaccharide production measured by Congo red staining. (4D) Swimming motility assay on semi-solid agar plates. (7A) GST pull-down identifying SfeP-interacting proteins from *Yptb* lysates or CHP-treated supernatants. (7C) Biofilm formation quantified by crystal violet staining after 20 h incubation in M9 medium. (7D) Extracellular polysaccharide production measured by Congo red assay. (7E-F) In vivo and in vitro GST pull-down assays confirming the SfeP–OmpF interaction by immunoblotting. (7G) Far-western assay showing binding of GST-OmpF to His-SfeP

Supplementary Material 2. Supplementary Tables: Table. S1 X-ray crystallography data collection and refinement statistics. Table. S2 Bacterial strains used in this study. Table. S3 Plasmids used in this study. Table. S4 Primers used in this study. Kristensen et al. (1995), Rosqvist et al. (1988), Hu et al. (2009) and Milton et al. (1996).

## Data Availability

All relevant data are within the manuscript and its Supporting Information files.

## Abbreviations

*Yptb*: *Yersinia pseudotuberculosis* YPIII
CP: Calprotectin
T6SS/1: Type VI secretion system /1
SfeP: T6SS-secreted ferrous iron-binding effector protein
Fe: Iron
Zn: Zinc
H_2_O_2_: Hydrogen peroxide
*P. aeruginosa*: *Pseudomonas aeruginosa*
*S*. Typhimurium: *Salmonella enterica* Serovar Typhimurium
*E. coli*: Escherichia coli
VSVG: Vesicular stomatitis virus G
EDDHA: Ethylenediamine-N, N′-bis [2-hydroxyphenylacetic acid]
EDDA: Ethylenediaminedi-[(o-hydroxyphenyl) acetic acid]
ASU: Asymmetric unit
Csp1: Copper storage protein 1
SlCsp3: *Streptomyces lividans* Copper storage proteins 3
RMSD: Root-mean-square deviation
IMEF: Iron-mineralizing encapsulin-associated Firmicute
WT: Wild-type
YLB: *Yersinia*-Lysogeny Broth
LB: Luria-Bertani broth
ITC: Isothermal titration calorimetry
PCR: Polymerase chain reaction
qRT-PCR: Quantitative Real-Time PCR
HE: Hematoxylin and eosin
CFU: Colony-forming units
BMDMs: Bone marrow derived macrophages
