# Supplementary material for: Yersinia pseudotuberculosis secretes an Fe (II)-binding effector to evade calprotectin-mediated nutritional immunity
Source: Stress Biol. 2026 Apr 14;6(1):29. doi: 10.1007/s44154-026-00304-6 (PMC13079257; doi:10.1007/s44154-026-00304-6)
Supplement: Supplementary file 1 — Supplementary Material 1. Supplementary Figures: Fig. S1 Growth curves of Yptb WT, ∆clpV1 mutant or complemented strain ∆clpV1(clpV1). Saturated bacterial cultures were diluted to fresh YLB medium. The growth of the cultures was monitored at indicated time points by measuring OD600. Fig. S2 SfeP is a Fe binding protein. (A) Top 10 threading templates of YPK_0411 predicted by I-TASSER. I-TASSER modeling starts from the structure templates identified by LOMETS from the PDB library. (B) Part of the residues of YPK_0411 and 6fwrA, the residues are colored in black, those residues in template which are identical to the residue in the query sequence are highlighted in color. (C) Phylogenetic relationship of Yptb YPK_0411 with homologous proteins in other bacteria. Different protein sequences were obtained from the SwissProt database. The phylogenetic tree was constructed using MEGA 6.0 by the neighbor-joining method and multiple sequence alignment was performed using CLUSTAL W. The scale bar indicates percentage of divergence (distance). SwissProt accession nos. of proteins from species are as follows: Xenorhabdus khoisanae (WP 348994227.1:1-92); Xenorhabdus khoisanae (WP 053067915.1:1-87); Xenorhabdus bovienii (WP 275366913.1:1-94); Y. pseudotuberculosis (YPK_0411); Y. pseudotuberculosis (WP 032466985.1:1-87); Salmonella enterica (EOF5965430.1:18-90); Pantoea ananatis (WP 264239268.1:3-95); Pantoea agglomerans (WP 277971703.1:3-95); Pseudomonas sp. (WP 369319847.1:45-138); Pseudomonas graminis (WP 083233100.1:50-142); Pseudomonas sp. (WP 401361070.1:1-95); Pseudomonas syringae (WP 122259395.1:1-88); Pseudomonas syringae pv. (EEB57359.1:20-107); Pseudomonas (WP 005771784.1:9-95); Methylococcales bacterium (MGH8550762.1:35-92); Agrobacterium vitis (WP 070149548.1:30-88); Myxococcota bacterium (MFC1482338.1:1-89); Streptomyces sp. (WP 269859640.1:3-89); Streptomyces sp. (WP 355860097.1:8-45); Streptacidiphilus jiangxiensis (WP 042442920.1:3-96); Streptomyces sp. (WP 3908578 [file 44154_2026_304_MOESM1_ESM.docx]

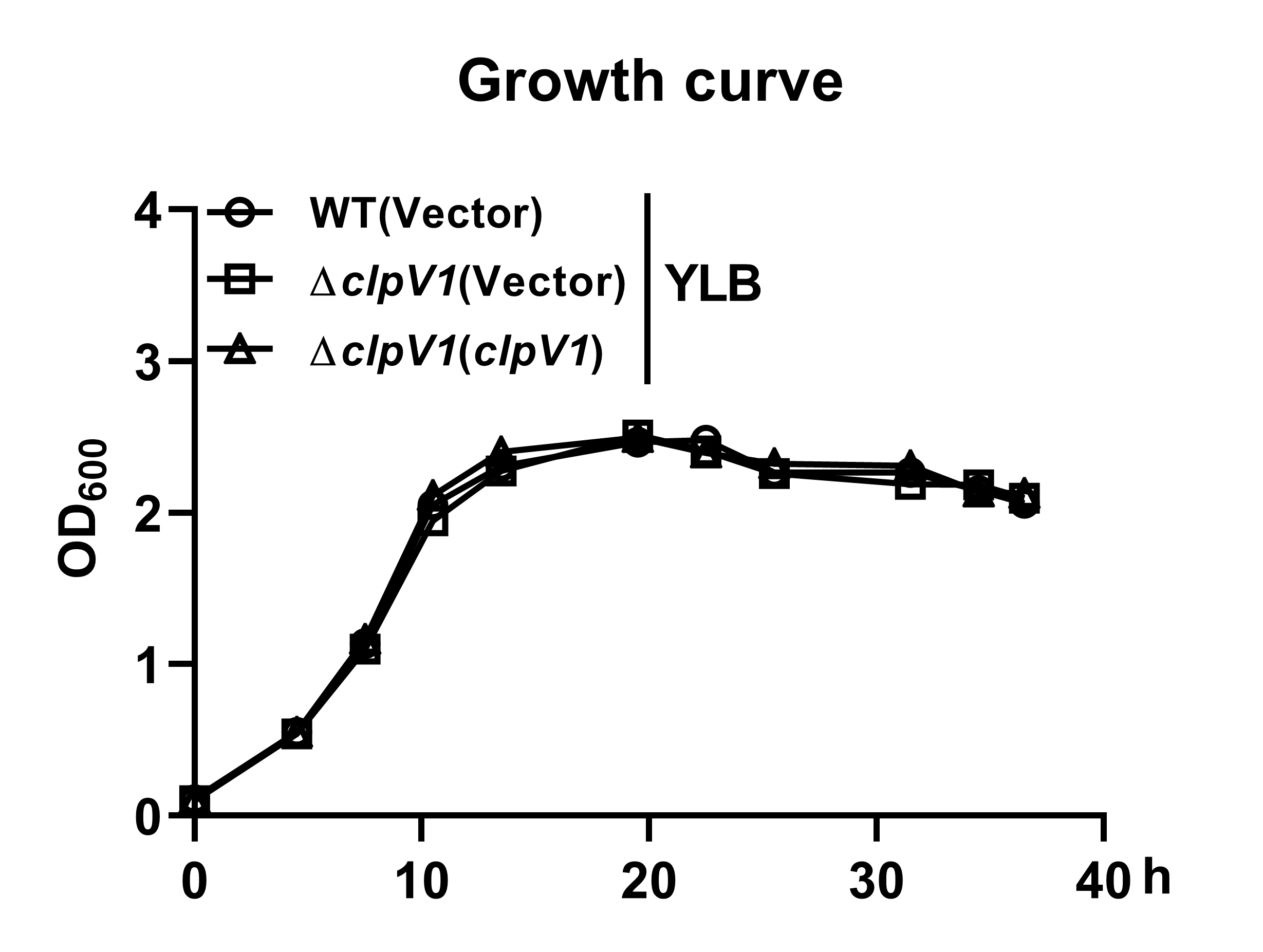


Fig. S1 Growth curves of *Yptb* WT, ∆*clpV1* mutant or complemented strain ∆*clpV1*(*clpV1*). Saturated bacterial cultures were diluted to fresh YLB medium. The growth of the cultures was monitored at indicated time points by measuring OD_600_.


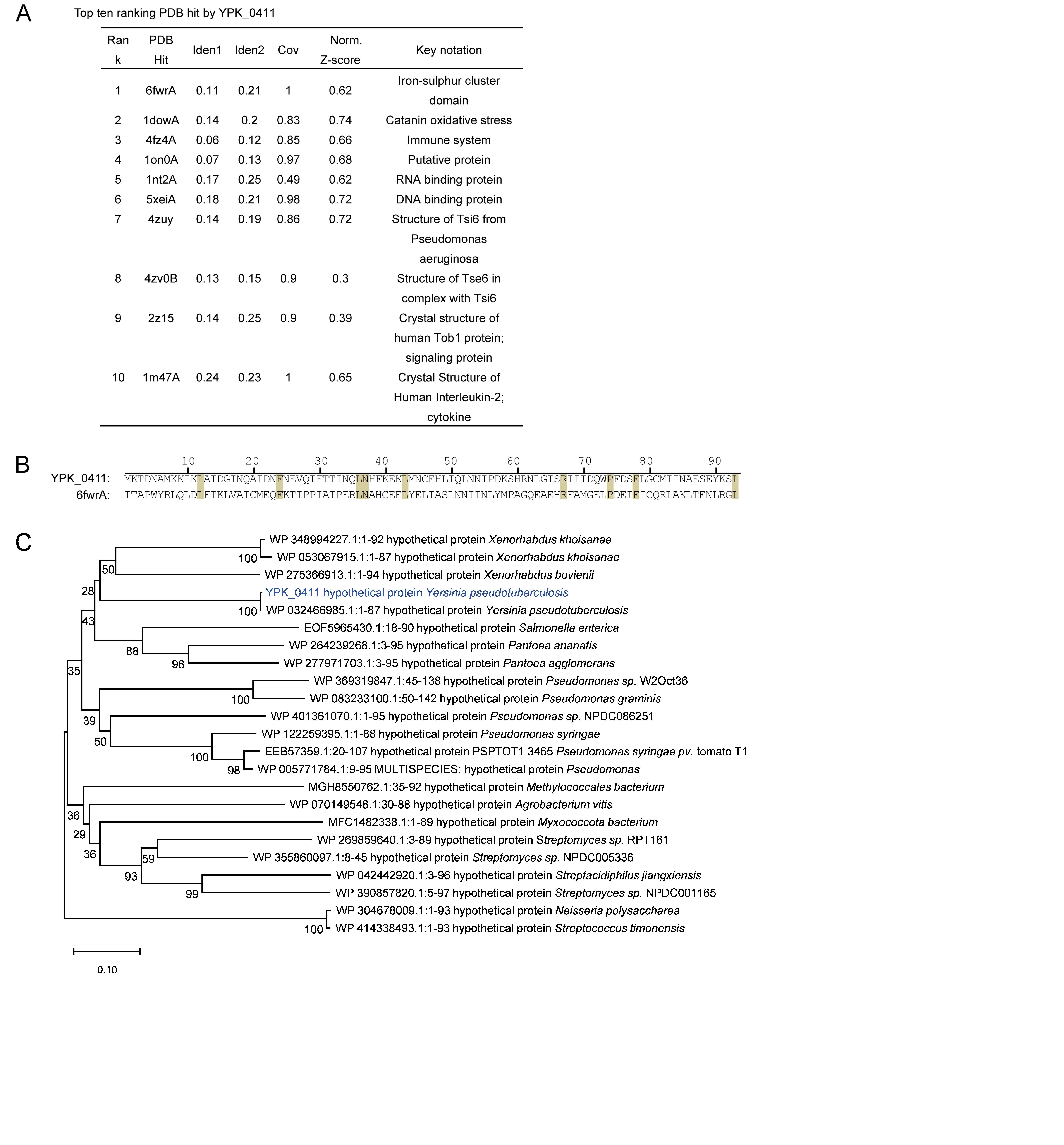


Fig. S2 SfeP is a Fe binding protein. (A) Top 10 threading templates of YPK_0411 predicted by I-TASSER. I-TASSER modeling starts from the structure templates identified by LOMETS from the PDB library. (B) Part of the residues of YPK_0411 and 6fwrA, the residues are colored in black, those residues in template which are identical to the residue in the query sequence are highlighted in color. (C) Phylogenetic relationship of *Yptb* YPK_0411 with homologous proteins in other bacteria. Different protein sequences were obtained from the SwissProt database. The phylogenetic tree was constructed using MEGA 6.0 by the neighbor-joining method and multiple sequence alignment was performed using CLUSTAL W. The scale bar indicates percentage of divergence (distance). SwissProt accession nos. of proteins from species are as follows: *Xenorhabdus khoisanae* (WP 348994227.1:1-92); *Xenorhabdus khoisanae* (WP 053067915.1:1-87); *Xenorhabdus bovienii* (WP 275366913.1:1-94); *Y. pseudotuberculosis* (YPK_0411); *Y. pseudotuberculosis* (WP 032466985.1:1-87); *Salmonella enterica* (EOF5965430.1:18-90); *Pantoea ananatis* (WP 264239268.1:3-95); *Pantoea agglomerans* (WP 277971703.1:3-95); *Pseudomonas sp.* (WP 369319847.1:45-138); *Pseudomonas graminis* (WP 083233100.1:50-142); *Pseudomonas sp.* (WP 401361070.1:1-95); *Pseudomonas syringae* (WP 122259395.1:1-88); *Pseudomonas syringae pv.* (EEB57359.1:20-107); *Pseudomonas* (WP 005771784.1:9-95); *Methylococcales bacterium* (MGH8550762.1:35-92); *Agrobacterium vitis* (WP 070149548.1:30-88); *Myxococcota bacterium* (MFC1482338.1:1-89); *Streptomyces sp.* (WP 269859640.1:3-89); *Streptomyces sp.* (WP 355860097.1:8-45); *Streptacidiphilus jiangxiensis* (WP 042442920.1:3-96); *Streptomyces sp.* (WP 390857820.1:5-97); *Neisseria polysaccharea* (WP 304678009.1:1-93); *Streptococcus timonensis* (WP 414338493.1:1-93).


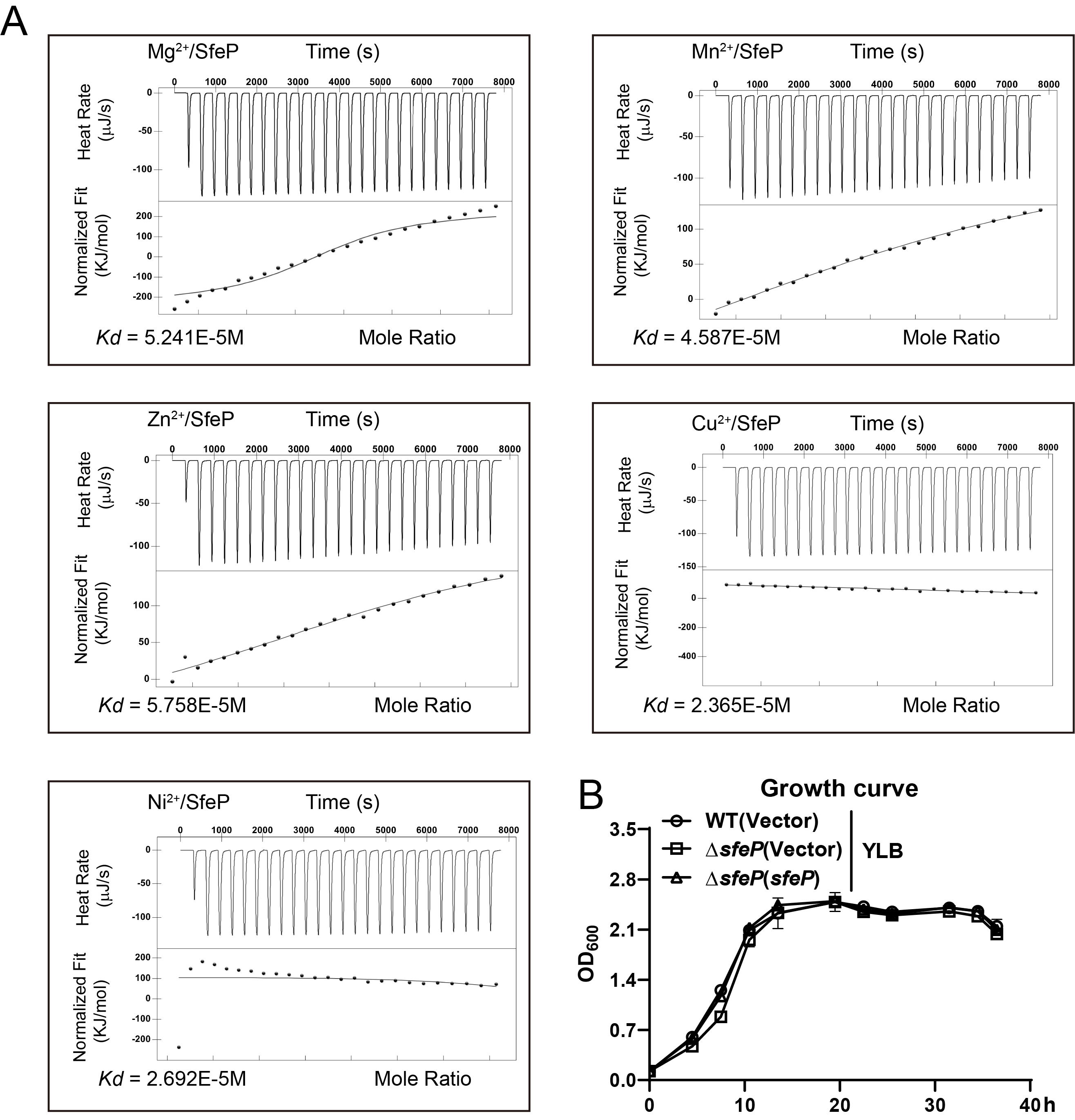


Fig. S3 SfeP is a ferrous iron binding protein. (A) The binding of Mg (II), Mn (II) Zn (II), Cu (II) and Ni (II) with SfeP protein was determined via ITC. Representative raw thermograms (upper panels) and the corresponding integrated heat plots with best-fit curves (lower panels) are shown. Heats of dilution (metal titrated into buffer alone) were subtracted prior to fitting. (B) Growth curves of *Yptb* WT, ∆*sfeP* mutant or complemented strain ∆*sfeP*(*sfeP*). Saturated bacterial cultures were diluted to fresh YLB medium. The growth of the cultures was monitored at indicated time points by measuring OD_600_.


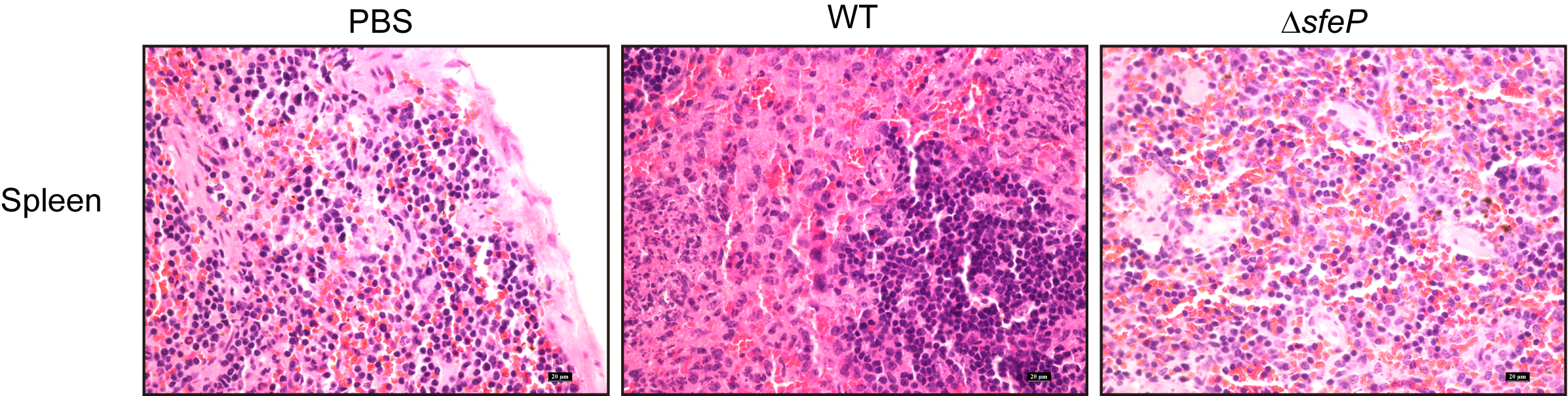


Fig. S4 SfeP is required for *Yptb* virulence. Hematoxylin-Eosin (HE) staining of the spleen of the C57BL/6 mice orogastrically inoculated with *Yptb* WT strain or ∆*sfeP* mutant. Tissues was collected at 72 h post-infection.


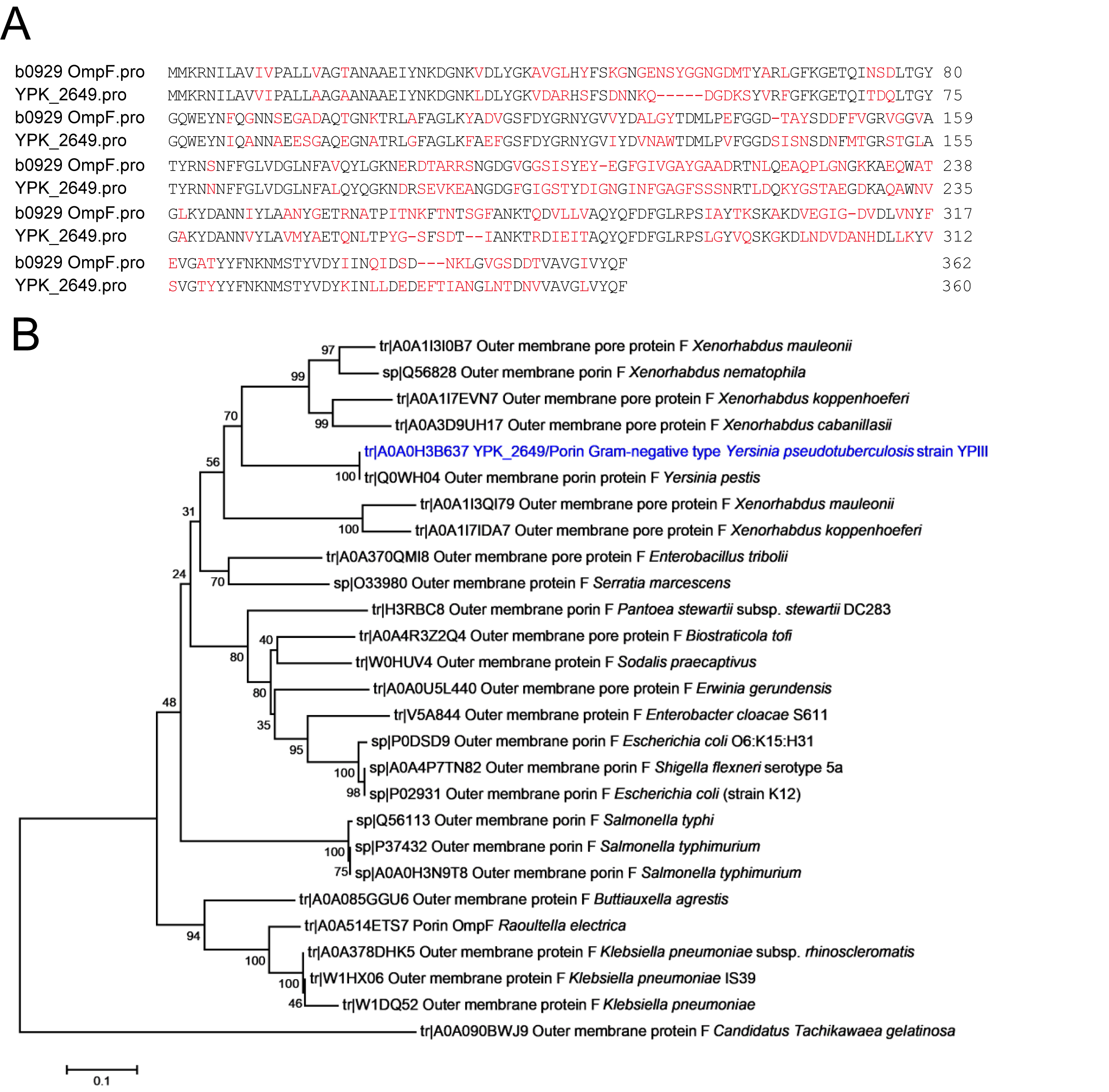


Fig. S5 The OmpF protein is widespread in bacteria. (A) Amino acid sequence similarity between *Escherichia coli* K-12 MG1655 OmpF (b0929) and *Yersinia pseudotuberculosis* OmpF (YPK_2649). The different residues are highlighted in red. (B) Phylogenetic relationship of *Yptb* OmpF with homologous proteins in other bacteria. Different protein sequences were obtained from the SwissProt database. The phylogenetic tree was constructed using MEGA 6.0 by the neighbor-joining method and multiple sequence alignment was performed using CLUSTAL W. The scale bar indicates percentage of divergence (distance). SwissProt accession nos. of proteins from species are as follows: *Y. pseudotuberculosis serotype* YPIII YPK_2649 (tr|A0A0H3B637); *Y. pestis* (tr|Q0WH04); *E. tribolii* (tr|A0A370QMI8); *X. mauleonii* (tr|A0A1I3I0B7); *X. koppenhoeferi* (tr|A0A1I7EVN7); *P. stewartii subsp. stewartii DC283* (tr|H3RBC8); *X. cabanillasii* (tr|A0A3D9UH17); Biostraticola tofi (tr|A0A4R3Z2Q4); *S. praecaptivus* (tr|W0HUV4); *E. gerundensis* (tr|A0A0U5L440); *X. mauleonii* (tr|A0A1I3QI79); *K. pneumoniae subsp. Rhinoscleromatis* (tr|A0A378DHK5); *K. pneumoniae IS39* (tr|W1HX06); *B. agrestis ATCC 33320* (tr|A0A085GGU6); *R. electrica* (tr|A0A514ETS7); *X. koppenhoeferi* (tr|A0A1I7IDA7); *E. coli O6:K15:H31* (sp|P0DSD9); *E. cloacae S611* (tr|V5A844); *S. flexneri serotype 5a* (sp|A0A4P7TN82); *S. typhi* (sp|Q56113); *K. pneumoniae* (tr|W1DQ52); *C. Tachikawaea gelatinosa* (tr|A0A090BWJ9); *S. typhimurium* (strain LT2) (sp|P37432); *S. typhimurium* (strain SL1344) (sp|A0A0H3N9T8); *E. coli* (strain K12) (sp|P02931); *X. nematophila* (sp|Q56828); *S. marcescens* (sp|O33980).


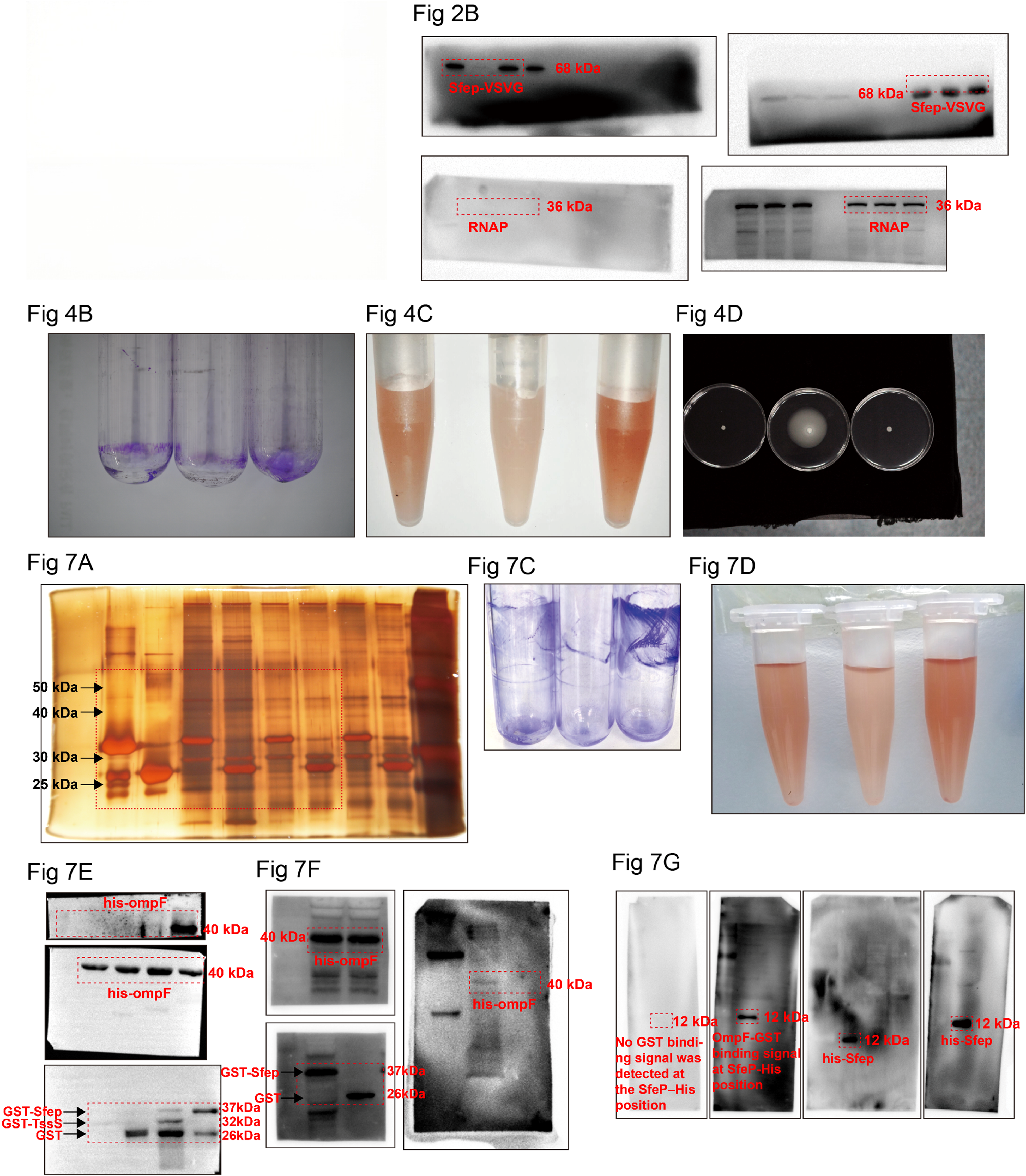


Fig. S6 The original images used in the manuscript. (2B) Immunoblot detection of SfeP in culture supernatants of the indicated strains. (4B) Biofilm formation on abiotic surfaces assessed by crystal violet staining. (4C) Extracellular polysaccharide production measured by Congo red staining. (4D) Swimming motility assay on semi-solid agar plates. (7A) GST pull-down identifying SfeP-interacting proteins from *Yptb* lysates or CHP-treated supernatants. (7C) Biofilm formation quantified by crystal violet staining after 20 h incubation in M9 medium. (7D) Extracellular polysaccharide production measured by Congo red assay. (7E-F) In vivo and in vitro GST pull-down assays confirming the SfeP–OmpF interaction by immunoblotting. (7G) Far-western assay showing binding of GST-OmpF to His-SfeP.
