# Supplementary material for: Yersinia pseudotuberculosis secretes an Fe (II)-binding effector to evade calprotectin-mediated nutritional immunity
Source: Stress Biol. 2026 Apr 14;6(1):29. doi: 10.1007/s44154-026-00304-6 (PMC13079257; doi:10.1007/s44154-026-00304-6)
Supplement: Supplementary file 2 — Supplementary Material 2. Supplementary Tables: Table. S1 X-ray crystallography data collection and refinement statistics. Table. S2 Bacterial strains used in this study. Table. S3 Plasmids used in this study. Table. S4 Primers used in this study. Kristensen et al. (1995), Rosqvist et al. (1988), Hu et al. (2009) and Milton et al. (1996). [file 44154_2026_304_MOESM2_ESM.docx]

Table. S1 X-ray crystallography data collection and refinement statistics.

| **Dataset** | **YPK_0411-Se** |
| --- | --- |
| **Data collection**  Beamline  Wavelength | BL-17U1, SSRF  0.979 |
| Resolution range* | 33.85-1.76(1.85-1.76) |
| Space group  Cell dimensions  a, b, c (Å)  α, β, γ (°) | *P*2_1_2_1_2_1_  29.86,47.39,67.69  90.00,90.00,90.00 |
| Total reflections | 125712(15715) |
| Unique reflections | 10026(1413) |
| Multiplicity | 12.5 |
| Completeness (%) | 99.9(100.0) |
| *Mean I/sigma(I)* | 14.0(4.0) |
| *R*-merge | 0.239(1.308) |
| *R*-meas | 0.250(1.375) |
| *R*-pim | 0.071(0.414) |
| CC1/2 | 0.980(0.815) |
| **Refinement** |  |
| Reflections used in refinement | 9965(956) |
| Reflections used for R-free | 449(49) |
| *R_work_* | 0.1990(0.3817) |
| *R_free_* | 0.2487(0.4619) |
| Wilson B-factor (Å^2^) | 6.55 |
| Number of non-hydrogen atoms | 918 |
| macromolecules | 763 |
| Protein residues | 93 |
| RMS(bonds) | 0.007 |
| RMS(angles) | 1.18 |
| Ramachandran favored (%) | 98.91 |
| Ramachandran allowed (%) | 1.09 |
| Ramachandran outliers (%) | 0.00 |
| Rotamer outliers (%) | 0.00 |
| Clashscore | 5.88 |
| Average B-factor(Å^2^) | 11.2 |

*For each structure one crystal was used.

*Values in parentheses are for the highest-resolution shell.

Table. S2 Bacterial strains used in this study.

| Strain | Characteristics | Source |
| --- | --- | --- |
| *Escherichia coli* |  |  |
| S17-1 λ pir | *λ*-pir lysogen of S17-1, *thi pro hsdR hsdM^+^ recA* RP4 2-Tc::Mu-Km::Tn7 | [^1^](#_ENREF_1) |
| BL21 (DE3) | Host for expression vector for pET28a | Novagen |
| *Yersinia pseudotuberculosis* |  |  |
| YPIII | Wild type *Yersinia pseudotuberculosis* pIB1 (*Yptb*), NaI^r^ | [^2^](#_ENREF_2) |
| ∆*fur* | *fur* gene deleted in *Yptb*, Nal^r^ | [^3^](#_ENREF_3) |
| ∆*clpV1* | *clpV1* gene deleted in *Yptb*, Nal^r^ | This study |
| ∆*sfeP* | *sfeP* gene deleted in *Yptb*, Nal^r^ | This study |
| ∆*ompF* | *ompF* gene deleted in *Yptb*, Nal^r^ | This study |
| ∆*sfeP*∆*ompF* | *sfeP* and *ompF* genes deleted in *Yptb*,  Nal^r^ | This study |
| YPIII (vector) | *Yptb* containing pKT100, Nal^r^, Km^r^ | This study |
| ∆*fur* (vector) | ∆*fur* containing pKT100, Nal^r^, Km^r^ | [^3^](#_ENREF_3) |
| ∆*fur* (*fur*) | ∆*fur* containing pKT100-*fur*, Nal^r^, Km^r^ | [^3^](#_ENREF_3) |
| ∆*clpV1* (vector) | ∆*clpV1* containing pKT100, Nal^r^, Km^r^ | This study |
| ∆*clpV1* (*clpV1*) | ∆*clpV1* containing pKT100-*clpV1*, Nal^r^, Km^r^ | This study |
| ∆*sfeP* (vector) | ∆*sfeP* containing pKT100, Nal^r^, Km^r^ | This study |
| ∆*sfeP* (*sfeP*) | ∆*sfeP* containing pKT100-*sfeP*, Nal^r^, Km^r^ | This study |
| ∆*ompF* (vector) | ∆*ompF* containing pKT100, Nal^r^, Km^r^ | This study |
| ∆*ompF* (*ompF*) | ∆*ompF* containing pKT100-*ompF*, Nal^r^,  Km^r^ | This study |
| ∆*sfeP*∆*ompF* (vector) | ∆*sfeP*∆*ompF* containing pKT100, Nal^r^,  Km^r^ | This study |
| ∆*sfeP*∆*ompF* (*sfeP*) | ∆*sfeP*∆*ompF* containing pKT100-*sfeP*, Nal^r^, Km^r^ | This study |
| ∆*sfeP*∆*ompF* (*ompF*) | ∆*sfeP*∆*ompF* containing pKT100-*ompF*, Nal^r^, Km^r^ | This study |

Table. S3 Plasmids used in this study.

| Plasmid |  |  |
| --- | --- | --- |
| pKT100 | Cloning vector, p15A replicon, Km^r^ | [^4^](#_ENREF_4) |
| pDM4 | Suicide vector, *mob*RK2, *ori*R6K, *pir*, *sacB*, Cm^r^ | [^5^](#_ENREF_5) |
| pET28a-*fur* | pET28a carrying fur coding region, Km^r^ | [^3^](#_ENREF_3) |
| pDM4-∆*clpV1* | Construct used for in-frame deletion of *clpV1*, Cm^r^ | This study |
| pDM4-T6SS-1*p::lacZ* | *T6SS-1::lacZ* fusion in pDM4 | This study |
| pKT100-*sfeP* | *sfeP* under the control of chloramphenicol resistance gene promoter in plasmid pKT100, Km^r^ | This study |
| pKT100-*ompF* | *ompF* under the control of chloramphenicol resistance gene promoter in plasmid pKT100, Km^r^ | This study |
| pET28a-*sfeP* | pET28a carrying *sfeP* coding region, Km^r^ | This study |
| pGEX-6p-1 | Expression vector with N-terminal GST tag, Amp^r^ | Novagen |
| pGEX6p-1-*sfeP* | pGEX6p-1 carrying *sfeP* coding region, Amp^r^ | This study |
| pET28a-*ompF* | pET28a carrying *ompF* coding region, Km^r^ | This study |
| pGEX6p-1-*ompF* | pGEX6p-1 carrying *ompF* coding region, Amp^r^ | This study |
| pDM4-*ompF* | Construct used for in-frame deletion of *ompF*, Cm^r^ | This study |
| pDM4-*sfeP* | Construct used for in-frame deletion of *sfeP* Cm^r^ | This study |

Table. S4 Primers used in this study.

| Primer | Sequence | Description |
| --- | --- | --- |
| *fur*-biotinF | TATCGCAATAAAAATGCCACTCATGTTTTAATATA | For EMSA, to generate T6SS1-p |
| *fur*-biotinR | TATATTAAAACATGAGTGGCATTTTTATTGCGATA |  |
| *fur*(sub)-biotinF | TATCGCAATCGGAACCAACGAAGAGCCGTAATATA | For EMSA, to generate T6SS1-pM |
| *fur*(sub)-biotinR | TATATTACGGCTCTTCGTTGGTTCCGATTGCGATA |  |
| T6P1F_NheI | ACCGGCTAGCACATTTGTTGGGTCAGAGTTA | pDM4-*T6SS-1p::lacZ* |
| T6P1R_XhoI | ACCGCTCGAGCATACTGATATGGTGCCAGTT |  |
| *clpV1*_M1F_BamHI | CGCGGATCCGGATCTGCAAAGCCCTGTATG | To generate pDM4-∆*clpV1* |
| *clpV1*_M1R | ATGGGGAAAAACTCGGGTAGTCGACGGTGCGCTATTAC |  |
| *clpV1*_M2F | TCGACGGTGCGCTATTAC |  |
| *clpV1*_M2R_SalI | ACGCGTCGACGTACCAGAGCCGAGTTTTCG |  |
| *clpV1*F_SalI | ACGCGTCGACATGATTCAAATTGACTTGCC | To generate pKT100-*clpV1* |
| *clpV*1R_BglII | GGAAGATCTTCATATCGCTCCCTCAAC |  |
| *sfeP*F-EcoRI | CCGGAATTCATGAAAACAGATAATGCAATGAAA | To generate pME6032-*sfeP-vsvg* |
| *sfeP*R-vsvg-TAA-XhoI | CCGCTCGAGTTATTTTCCTAATCTATTCATTTCAATATCTGTATAAAGACTTTTATATTCTGACTCGGC |  |
| *sfeP*M1F_BglII | GGAAGATCTATGAGCAGGGGATCCACT | To generate pDM4-∆*sfeP* |
| *sfeP*M1R | GCTTGATTAATTCCATCTATAGC |  |
| *sfeP*M2F | GCTATAGATGGAATTAATCAAGCGCCGAGTCAGAATATAA |  |
| *sfeP*M2R_SalI | ACGCGTCGACATATCCCCCGAGCCAAAC |  |
| *sfeP*_FBamHI | CGCGGATCCATGAAAACAGATAATGCAATG | To generate *sfeP* ORF |
| *sfeP*_RSalI | ACGCGTCGACTTAAAGACTTTTATATTCTGACTC |  |
| *ompF*M1F_BglII | GGAAGATCTTCAGGTTGACTACACTGATGCC | To generate pDM4-∆*ompF* |
| *ompF*M1R | AGAAAGAGTGACGTGCGTCA |  |
| *ompF*M2F | TGACGCACGTCACTCTTTCTTGCTGGACGAAGATGAGTTC |  |
| *ompF*M2R_SalI | ACGCGTCGACCCGTGGAACAATACCACATC |  |
| *ompF*F_SalI | ACGCGTCGACATGATGAAGCGCAATATTCTT | To generate *ompF* ORF |
| *ompF*R_BglII | GGAAGATCTTTAGAACTGGTAAACCAAGCC |  |
| *16s*_F | CCTTCGGGTTGTAAAGCA | qRT-PCR |
| *16s*_R | TCCGATTAACGCTTGCAC | qRT-PCR |
| *clpV1*_F | CAGCAGCGAATTGAGATCG | qRT-PCR |
| *clpV1*_R | ATCTGTGGCGCTCTCTTCTT | qRT-PCR |
| *hcp1*_F | ACATTGTGACGGTGCCTACC | qRT-PCR |
| hcp1_R | GGTGAAATGCTGCCAACAG | qRT-PCR |
| *icmF*_F | CAACTGCTGGAAGGCATT | qRT-PCR |
| *icmF*_R | AAGGTGATATTGCACCGC | qRT-PCR |
| *vgrG*_F | AGGCGCAGGAACTGAGTAT | qRT-PCR |
| vgrG_R | TTTGCAGTTCTCCCGACT | qRT-PCR |
| *sfeP*_F | TAGCTATAGATGGAATTAATCAAGC | qRT-PCR |
| *sfeP*_R | ATTTGTCAGGGATGTTATTTAGTTG | qRT-PCR |
| *gapdh*_F | CGACTTCAACAGCAACTCCCACTCTTCC | qRT-PCR |
| *gapdh* _R | TGGGTGGTCCAGGGTTTCTTACTCCTT | qRT-PCR |
| *s100a8*_F | TGTCCTCAGTTTGTGCAGAATATAAA | qRT-PCR |
| *s100a8*_R | TCACCATCGCAAGGAACTCC | qRT-PCR |
| *s100a9*_F | GGTGGAAGCACAGTTGGCA | qRT-PCR |
| *s100a9*_R | GTGTCCAGGTCCTCCATGATG | qRT-PCR |

References

1. Kristensen, C.S., Eberl, L., Sanchez-Romero, J.M., Givskov, M., Molin, S., and De Lorenzo, V. Site-specific deletions of chromosomally located DNA segments with the multimer resolution system of broad-host-range plasmid RP4. J Bacteriol. 1995;177, 52-58.

2. Rosqvist, R., Skurnik, M., and Wolf-Watz, H. Increased virulence of Yersinia pseudotuberculosis by two independent mutations. Nature. 1988;334, 522-524.

3. Wang, T., Si, M., Song, Y., Zhu, W., Gao, F., Wang, Y., et al. Type VI Secretion System Transports Zn^2+^ to Combat Multiple Stresses and Host Immunity. PLoS Pathog. 2015;11, e1005020.

4. Hu, Y., Lu, P., Wang, Y., Ding, L., Atkinson, S., and Chen, S. OmpR positively regulates urease expression to enhance acid survival of Yersinia pseudotuberculosis. Microbiology (Reading). 2009;155, 2522-2531.

5. Milton, D.L., O'Toole, R., Horstedt, P., and Wolf-Watz, H. Flagellin A is essential for the virulence of Vibrio anguillarum. J Bacteriol. 1996;178, 1310-1319.
